# Booster with Ad26.COV2.S or Omicron-adapted vaccine enhanced immunity and efficacy against SARS-CoV-2 Omicron in macaques

**DOI:** 10.1038/s41467-023-37715-2

**Published:** 2023-04-07

**Authors:** Laura Solforosi, Lea M. M. Costes, Jeroen T. B. M. Tolboom, Katherine McMahan, Tochi Anioke, David Hope, Tetyana Murdza, Michaela Sciacca, Emily Bouffard, Julia Barrett, Cindy Wu, Nicole Hachmann, Jessica Miller, Jingyou Yu, Xuan He, Catherine Jacob-Dolan, Sietske K. Rosendahl Huber, Liesbeth Dekking, Ronnie Chamanza, Ying Choi, Karin Feddes-de Boer, Dan H. Barouch, Hanneke Schuitemaker, Roland C. Zahn, Frank Wegmann

**Affiliations:** 1 Janssen Vaccines and Prevention B.V., Leiden, Netherlands; 2 Center for Virology and Vaccine Research, Beth Israel Deaconess Medical Center, Harvard Medical School, Boston, MA USA; 3 Non-Clinical Safety Toxicology/Pathology, Janssen Research and Development, Beerse, Belgium; 4 Ragon Institute of MGH, MIT and Harvard, Cambridge, MA USA; 5 Harvard Medical School, Boston, MA USA; 6 Massachusetts Consortium on Pathogen Readiness, Boston, MA USA

**Keywords:** Infectious diseases, Diseases, SARS-CoV-2, Vaccines

## Abstract

Omicron spike (S) encoding vaccines as boosters, are a potential strategy to improve COVID-19 vaccine efficacy against Omicron. Here, macaques (mostly females) previously immunized with Ad26.COV2.S, are boosted with Ad26.COV2.S, Ad26.COV2.S.529 (encoding Omicron BA.1 S) or a 1:1 combination of both vaccines. All booster vaccinations elicit a rapid antibody titers increase against WA1/2020 and Omicron S. Omicron BA.1 and BA.2 antibody responses are most effectively boosted by vaccines including Ad26.COV2.S.529. Independent of vaccine used, mostly WA1/2020-reactive or WA1/2020-Omicron BA.1 cross-reactive B cells are detected. Ad26.COV2.S.529 containing boosters provide only slightly higher protection of the lower respiratory tract against Omicron BA.1 challenge compared with Ad26.COV2.S-only booster. Antibodies and cellular immune responses are identified as complementary correlates of protection. Overall, a booster with an Omicron-spike based vaccine provide only moderately improved immune responses and protection compared with the original Wuhan-Hu-1-spike based vaccine, which still provide robust immune responses and protection against Omicron.

## Abstract (web summary)

Variant booster vaccines are a strategy to improve protection against SARS-CoV-2. Here, the authors find that both Wuhan-Hu-1-based Ad26.COV2.S or an Omicron-adapted booster vaccine provide robust immune responses and protection against Omicron in NHP.

## Introduction

The emergence of SARS-CoV-2 variants of concern (VoC) poses a risk for the protective efficacy of COVID-19 vaccines based on the ancestral Wuhan-Hu-1 Spike (S). This is due to arising of mutations in the virus S glycoprotein, associated with partial evasion from (humoral) immunity against earlier S variants elicited by natural viral exposure or vaccination^1–3^ and increased transmissibility and virulence in humans^4–6^.

The emergence of Omicron BA.1(initially named B.1.1.529) and Omicron subvariants (BA.2, BA.4, BA.5, BA2.75^4,7–9^) has increased the concern around vaccine efficacy, as they are the genetically most distant VoCs described so far, with more than 30 amino acidic substitutions in S, 15 of which located in the receptor binding domain (RBD)^10^, the main target of neutralizing antibodies. Neutralization capacity induced by passive immunization with therapeutic antibodies or actively elicited by vaccines based on the ancestral Wuhan-Hu-1 SARS-CoV-2 strain or infection, is reduced to a greater extent against variants carrying these mutations compared with S substitutions associated with earlier SARS-CoV-2 VoCs^8,11–16^. A booster immunization with the first generation, Wuhan-based vaccines, has been shown to augment Omicron-specific neutralizing antibody responses in humans^13,17^ and NHP models^18^, however, antibody levels wane over time, depending on the vaccine platform^19^, and periodic boosters are expected to be required to maintain vaccine efficacy against newly emerging VoCs^20–23^. Hence, COVID-19 vaccines matching S of VoCs have been considered as a strategy to elicit a more specific immune response against VoCs^24,25^. Recently, based on immunogenicity data, mRNA vaccines that include an Omicron S encoding component have been authorized for human use in the United States (US)^26^, Europe^27^ and United Kingdom (UK)^28^, although efficacy data are not yet available.

A single dose of Ad26.COV2.S demonstrated an efficacy of 74.6% against severe-critical COVID-19, 75.6% against COVID-19 leading to medical intervention (including hospitalization), and 82.8% against COVID-19-related death^29^, in a phase 3 clinical trial that included the emergence of the Beta (B.1.351) variant in South Africa. A 2-dose Ad26.COV2.S regimen with 8 weeks interval, showed a global efficacy of 75.2% against moderate to severe–critical COVID-19 and 100% against severe–critical COVID 19, in a phase 3 clinical trial where most cases were due to the variants alpha (B.1.1.7) and mu (B.1.621)^30^. In addition, a real-world evidence study showed that a homologous booster with Ad26.COV2.S administered 6-9 months after primary single dose vaccination provided more than 80% protection against hospitalization during the Omicron wave in South Africa^31^.

Here we report immunogenicity and efficacy of a booster vaccination with Ad26.COV2.S, or an experimental variant vaccine encoding Omicron BA.1 spike (Ad26.COV2.S.529), or the combination of the two vaccines, against SARS-CoV-2 BA.1 in non-human primates (NHP) that had received Ad26.COV2.S vaccination about twenty months earlier.

## Results

### Booster vaccination with Ad26.COV2.S, Ad26.COV2.S.529 or the vaccine combination induced a rapid and robust increase of humoral immune responses in NHPs previously immunized with Ad26.COV2.S

**Fig. 1:**
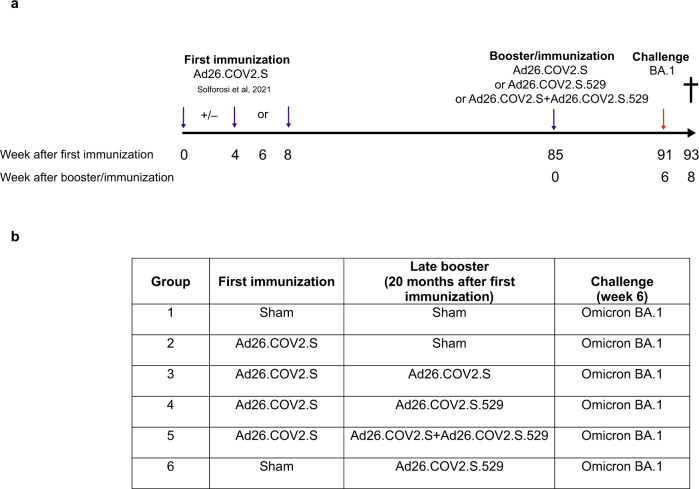
Study design. **a** Primary and booster vaccine regimens and the timing of immunization and challenge are shown. Blue arrows indicate immunizations. The red arrow indicates virus inoculation. The cross indicates sacrifice. **b** Tabular study design.

Adult Chinese-origin rhesus macaques (*Macaca mulatta*, *n* = 28) previously immunized with a single or 2-dose Ad26.COV2.S regimen^32^, were assigned to 4 groups by a randomizing stratification system based on Wuhan S binding and neutralizing antibody levels, previous immunization regimen, body weight and age. For the present study, these NHPs received either a booster immunization with 5 × 10^10^ viral particles (vp) Ad26.COV2.S (*n* = 7), 5 × 10^10^ vp Ad26.COV2.S.529 (*n* = 7), the combination of the 2 vaccines at a total dose of 5 × 10^10^ vp (*n* = 7) or no booster (*n* = 7), twenty months (week 85) after the primary vaccination regimen (Fig. 1). The study also included a group of naïve sham control NHPs (n = 8) that received an injection with saline, and a group of naïve NHPs (*n* = 6) that received a single dose of 5 × 10^10^ vp Ad26.COV2.S.529. Blood samples were collected before the booster/immunization and at weeks 1, 2, 4 and 6 after the booster/immunization to measure binding and neutralizing antibody levels. At week 6 after the booster/immunization, all animals were challenged with SARS-CoV-2 Omicron (BA.1) and additional blood samples were collected 1 and 2 weeks post-challenge.

**Fig. 2:**
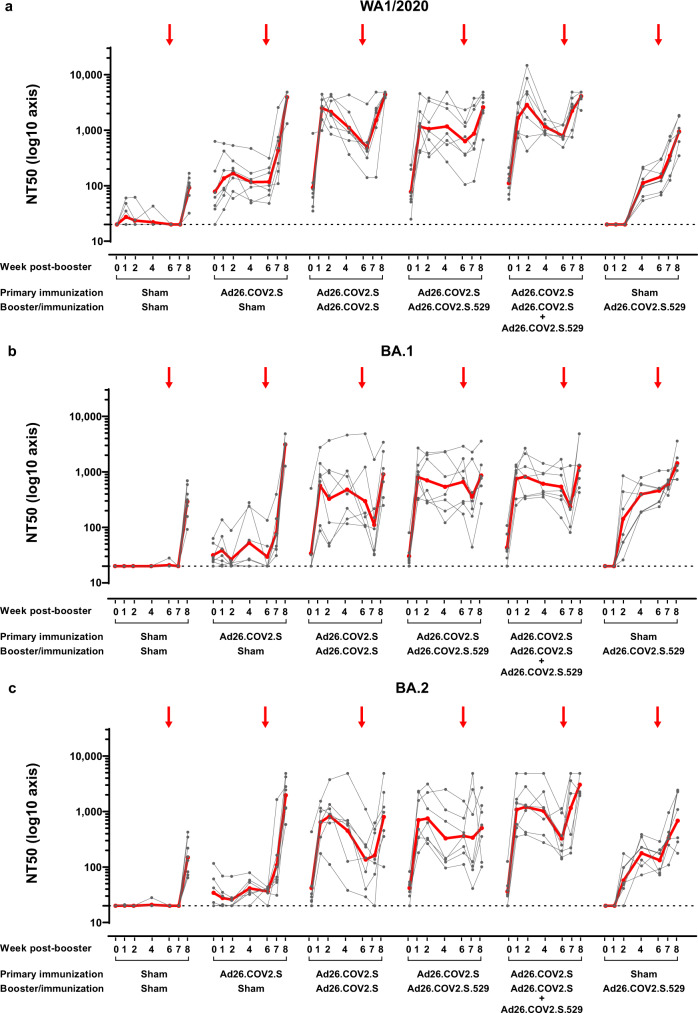
SARS-CoV-2 S WA1/2020, BA.1-, and BA.2-specific neutralizing antibody responses to Ad26.COV2.S, Ad26.COV2.S.529, or a combination of Ad26.COV2.S and Ad26.COV2.S.529 late booster in adult rhesus macaques. SARS-CoV-2 neutralizing antibody titers were measured over time in NHP serum samples (42 NHPs and 7 time points) with a luciferase-based psVNA specific for the S glycoprotein of SARS-CoV-2 of the **a** WA1/2020 strain (Wuhan/WIV04/2019, GISAID accession ID: EPI_ISL_402124 [https://gisaid.org/wiv04/]), **b** Omicron BA.1 strain (GISAID accession ID: EPI_ISL_7358094.2 [https://gisaid.org]), and **c** Omicron BA.2 strain (GISAID accession ID: EPI_ISL_6795834.2 [https://gisaid.org]). Neutralizing antibody responses were expressed as the reciprocal of the sample dilution where 50% neutralization was achieved (NT50). Neutralizing antibody levels in individual animals are depicted with gray points and paired measurements connected with gray lines. The GMT of neutralizing antibody responses per group is indicated with the red line. The horizontal dotted line indicates the LLOD. The red arrows indicate the time of challenge (week 6). For all panels, *n* = 7 animals per group except for the Sham-Ad26.COV2.S.529 group where *n* = 6 animals and for the Sham-Sham group where *n* = 8 animals. Comparisons between specific vaccine groups were made in a Tobit ANOVA with a post hoc z- or t-test. Source data are provided as a Source Data file. ANOVA, analysis of variance; GMT, geometric mean titer; LLOD, lower limit of detection; NHP, nonhuman primate; NT50, 50% neutralization titer; psVNA, pseudovirus neutralization assay; S, spike.

Neutralizing antibody responses were measured by luciferase-based pseudovirus neutralization assays (psVNA). At the pre-booster timepoint, vaccine-matched WA1/2020 S neutralizing antibody titers were still detectable in previously Ad26.COV2.S immunized NHPs (groups 2-5, Fig. 1) with a geometric mean titer (GMT) of 89 50% neutralization titer (NT50) (Fig. 2a). These titers showed a 2- to 5-fold decay, depending on the vaccine regimen, compared with the previously reported titers at week 14 after the primary immunization^32^ (Supplementary Fig. 1a). Low levels of Omicron BA.1- and BA.2-specific neutralizing antibody titers were measurable as well, with GMT of 34 and 38 NT50, respectively, slightly above the lower limit of detection (LLOD = 20) (Fig. 2b and c). Regardless of the booster vaccine applied, a rapid and robust increase of approximately 20-fold of WA1/2020, BA.1 and BA.2 neutralizing antibody titers was observed, as measured 1 week after the booster (Fig. 2). Following the post-booster peak responses at week 1-2, Omicron BA.1 neutralizing antibody titers showed only a modest decline up to the time of challenge (week 6) and were comparable at all measured time points among the differently boosted groups (Fig. 2b). WA1/2020 and BA.2 neutralizing antibody responses showed a more rapid decline after peak responses, particularly when elicited by Ad26.COV2.S or the vaccine combination (Fig. 2a and c). At week 6 post-booster, the BA.2 neutralizing antibody GMT elicited by a booster with Ad26.COV2.S.529 or the vaccine combination was 2.65- (*p* = 0.021, Tobit analysis of variance [ANOVA] z -test) or 2.39-fold higher (*p* = 0.039, Tobit ANOVA z test), respectively, compared with the GMT elicited by the Ad26.COV2.S booster (Fig. 2c). WA1/2020 neutralizing antibody GMT elicited by Ad26.COV2.S, at week 1 post-booster, were 2.15-fold higher (*p* = 0.05, Tobit ANOVA z test) compared with GMTs elicited by Ad26.COV2.S.529 (Fig. 2a).

In naïve NHP immunized with a single dose of Ad26.COV2.S.529, BA.1 and BA.2 neutralizing antibodies were measurable starting from week 2 post-immunization and further increased by week 4 (Fig. 2b and c), showing similar kinetics as WA1/2020 antibody responses elicited by Ad26.COV2.S in naïve rhesus macaques^32,33^. By week 6 post-immunization, the BA.1 neutralizing antibody GMTs were comparable with the GMTs reached in boosted animals. The BA.2 neutralizing antibody GMT were 2.74- (*p* = 0.022, Tobit ANOVA z test) and 2.47-fold lower (*p* = 0.04, Tobit ANOVA z test) compared with animals receiving a booster with Ad26.COV2.S.529 or the vaccine combination, and comparable to titers in animals boosted with Ad26.COV2.S. In naïve NHP, Ad26.COV2.S.529 also elicited WA1/2020 neutralizing antibodies, measurable starting from week 4 post-immunization. However, at week 6, the magnitude of these responses was 3.38- to 5.47-fold lower (*p* ≤ 0.002, ANOVA t test) compared with animals that were previously immunized with Ad26.COV2.S (Fig. 2a).

RBD-specific binding antibodies were measured by enzyme-linked immunosorbent assay (ELISA). As observed for neutralizing antibodies, vaccine-matched WA1/2020 S-binding antibody titers were still detectable at the pre-booster timepoint in previously Ad26.COV2.S immunized NHPs, (GMT of 564; Supplementary Fig. 2a). Compared with the reported titers at week 14 after primary immunization^32^ these titers decreased between 2- to 4-fold depending on the vaccine regimens (Supplementary Fig. 1b). Also Omicron BA.1 and BA.2 S binding antibody titers were detected at the pre-booster timepoint (GMT of 128 and 141, respectively; Supplementary Fig. 2b and c). Post-booster, the kinetics of binding antibody responses were comparable to neutralizing antibodies, with a steep 40- to 70-fold increase of antibody levels within 1 week in all boosted animals depending on the antigen specificity (Supplementary Fig. 2). At week 6 post-booster, the RBD BA.1 binding antibody GMT elicited by a booster with Ad26.COV2.S.529 or the vaccine combination was 2.42- (*p* = 0.027, ANOVA t-test) or 2.61-fold higher (*p* = 0.017, ANOVA t-test), respectively, compared with GMT elicited by Ad26.COV2.S booster (Supplementary Fig. 2). BA.2 binding antibody titers measured at week 2 and week 4 post-immunization were 2.27- (*p* = 0.009, ANOVA t-test) and 2.52-fold higher (*p* = 0.025, ANOVA t-test), respectively, in animals immunized with the vaccine combination compared with Ad26.COV2.S. WA1/2020 binding antibody titers were comparable at all time points measured among the different boosted groups.

In naïve NHPs immunized with a single dose of Ad26.COV2.S.529, RBD WA1/2020, BA.1, and BA.2 binding antibodies were measurable starting from week 2 post-immunization, further increased by week 4 and stabilized at week 6 (Supplementary Fig. 2). At week 6 post-immunization, the magnitude of BA.1 binding antibody responses was similar to the antibody levels in preimmunized animals boosted with Ad26.COV2.S.529 and the vaccine combination, while they were 2.56-fold higher (*p* = 0.024, ANOVA t-test) than antibody levels in Ad26.COV2.S-boosted animals (Supplementary Fig. 2b). Similar observations were made for RBD BA.2 specific antibodies, while RBD WA1/2020 specific antibodies were generally lower than for boosted animals (Supplementary Fig. 2a).

**Fig. 3:**
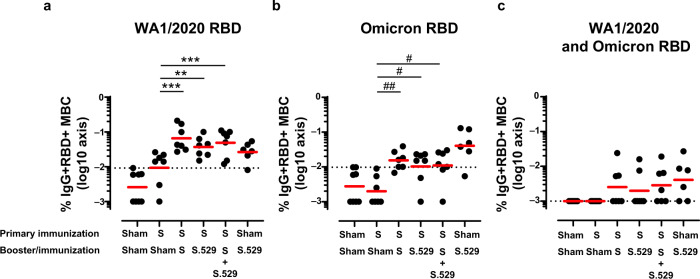
RBD-specific memory B-cell responses following vaccination. Frequency of **a** WA1/2020, **b** Omicron BA.1, and **c** cross-reactive WA1/2020 and Omicron BA.1 RBD-specific memory B-cell responses measured in 42 NHP PBMC samples by flow cytometry 6 weeks after the late booster/immunization. The GM response per group is indicated with the red horizontal line. The y-axis was log10 transformed for better visualization. The dotted line indicates the positivity threshold, calculated as the 95th percentile of sham responses. For all panels, values equal to 0 were imputed to 0.001 for visualization purposes. For all panels, *n* = 7 animals per group except for the Sham-S.529 group where *n* = 6 animals and for the Sham-Sham group where *n* = 8 animals. Comparisons between the sham-immunized group and all other groups with 5-fold Bonferroni adjustment as well as pairwise comparisons between all groups except the control sham-immunized group were made with a 2-sided t-test or exact Mann-Whitney U-test. T-test: **p < 0.01; ***p < 0.001. Mann-Whitney U-test: ^#^*p* < 0.05; ^##^*p* < 0.01. Non^-^significant comparisons are not indicated on the graphs. The complete statistical analysis is reported in Supplementary Table 1. Source data are provided as a Source Data file. GM geometric mean, MBC memory B cell, NHP non-human primate, PBMC peripheral blood mononuclear cell, RBD receptor-binding domain, S spike.

Naïve animals or non-boosted animals did not develop antibody responses or show changes in antibody levels, respectively, up to 1 week after the Omicron BA.1 challenge (week 7) (Fig. 2 and Supplementary Fig. 2). Following Omicron BA.1 challenge, an increase of S-neutralizing and RBD-binding antibodies was generally observed in all groups (Fig. 2 and Fig. 3). However, neutralizing antibodies specific for the challenge virus Omicron BA.1 showed a transient decrease in titer one week after challenge in boosted animals, that increased again at later time points (Fig. 2b).

Breadth of antibody responses against the major VOCs was evaluated in multiplex S- and RBD-specific binding assays using the Meso Scale Discovery electrochemiluminescence assay (ECLA) platform^34^. A booster immunization with Ad26.COV2.S, Ad26.COV2.S.529 or the vaccine combination induced comparable antibody levels against Wuhan, Alpha, Beta, Gamma, Delta and Omicron S and RBD at 2 weeks post-booster. A single dose of Ad26.COV2.S.529 administered to naïve NHPs, elicited binding antibodies against all tested heterologous VOCs S and RBD (Supplementary Fig. 3).

### Booster vaccination with Ad26.COV2.S, Ad26.COV2.S.529 or the vaccine combination augmented cellular SARS-CoV-2 specific immune responses

Six weeks post-vaccination, antigen-specific memory immunoglobulin G (IgG)+ B cells in peripheral blood were measured using multiparameter flow cytometry. In Ad26.COV2.S pre-immunized boosted animals, the levels of memory B cells were comparable among groups, independent of the booster vaccination they received, and higher compared with non-boosted animals (*p* ≤ 0.008, Tobit ANOVA z-test for WA1/2020 responses, and *p* ≤ 0.021, Mann-Whitney test for Omicron BA.1 responses), indicating an expansion of memory B cells following the booster (Fig. 3). In non-boosted NHPs, low levels of WA1/2020 RBD-specific memory B cells were still detectable twenty months after the primary vaccination. In boosted animals most of the detected memory B cells were WA1/2020 RBD-reactive or cross-reactive to WA1/2020 and Omicron BA.1 RBD (Fig. 3a and c), and there was a limited number of exclusively Omicron BA.1 RBD-specific memory B cells (Fig. 3b). In naïve NHPs immunized with a single dose of Ad26.COV2.S.529, Omicron BA-1 RBD-reactive memory B cells were detected at a higher level compared with boosted animals (Fig. 3b) and were slightly higher than WA1/2020 RBD-specific B cells (Fig. 3a and b). In these animals, cross-reactive memory B cells (Fig. 3c) were detectable as well. These data indicate that in previously Ad26.COV2.S immunized rhesus macaques, a booster vaccination with Ad26.COV2.S, Ad26.COV2.S.529 or the vaccine combination mostly elicited an expansion of WA1/2020-reactive, and WA1/2020 and Omicron BA.1 cross-reactive RBD-specific memory B cells. These data are consistent with the rapid and robust antibody responses against WA1/2020 and Omicron antigens measured post-booster (Fig. 2 and Supplementary Fig. 2).

**Fig. 4:**
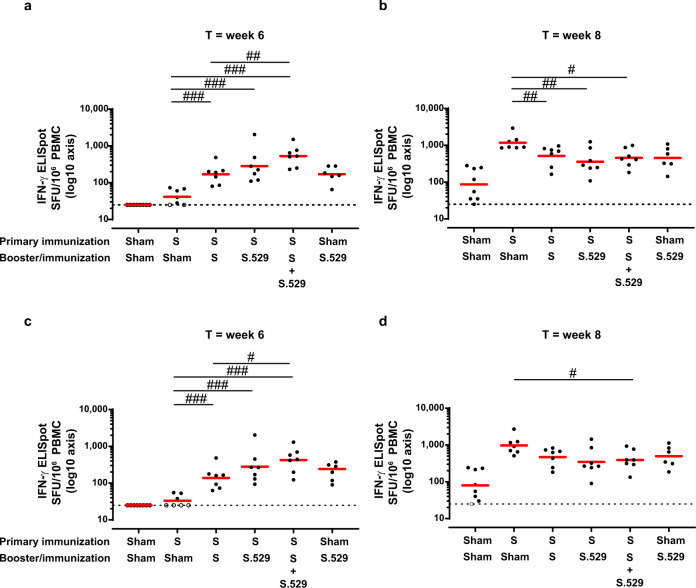
SARS-CoV-2–specific cellular immune responses after Ad26.COV2.S, Ad26.COV2.S.529, or a combination of Ad26.COV2.S and Ad26.COV2.S.529 late booster in adult rhesus macaques. WA1/2020 (**a** and **b**) and Omicron BA.1 (**c** and **d**) S-specific T cell responses, as measured in 42 NHP PBMC samples with an IFN-γ ELISpot 6 weeks after the booster/immunization (**a** and **c**) and 2 weeks post-challenge with Omicron BA.1. PBMCs were either non-stimulated (i.e., medium stimulated representing background) or stimulated with S WA1/2020 or Omicron BA.1. Background-subtracted counts for individual NHPs are indicated by a dot. The GM response per group is indicated with the red horizontal line. Samples with background-subtracted counts ≤25 were set at 25 for visualization purposes and indicated by open symbols and the dotted line. For all panels, *n* = 7 animals per group except for the Sham-S.529 group where *n* = 6 animals and for the Sham-Sham group where *n* = 8 animals. Comparisons between the sham-immunized group and all other groups with 5-fold Bonferroni adjustment as well as pairwise comparisons between groups except the control sham-immunized group were made with a 2-sided z-test, t-test, or exact Mann-Whitney U-test. ^#^*p* < 0.05; ^##^*p* < 0.01; ^###^*p* < 0.001. Non-significant comparisons are not indicated on the graphs. The complete statistical analysis is reported in Supplementary Table 2. Source data are provided as a Source Data file. ELISpot enzyme-linked immunospot assay, GM geometric mean, IFN-γ interferon gamma, NHP nonhuman primate, PBMC peripheral blood mononuclear cell, S spike, SFU spot-forming units, T timepoint.

S-specific T cell responses elicited by vaccination were measured by Interferon-gamma (IFN-γ) Enzyme-Linked ImmunoSpot assay (ELISpot) and intracellular cytokine staining (ICS). In about half of the non-boosted animals, low levels of T cell responses were still detectable twenty months after the primary vaccination by ELISpot (Fig. 4a and c). Ad26.COV2.S pre-immunized animals that received a late booster with Ad26.COV2.S, Ad26.COV2.S.529 or the vaccine combination, had higher numbers of WA1/2020- and Omicron BA.1-specific IFN-γ producing cells, as measured six weeks post-booster, compared with animals that did not receive a booster immunization (*p* < 0.001, Mann-Whitney test), indicating that T cell responses were expanded by the late booster vaccination (Fig. 4a and c). Animals boosted with the vaccine combination had the highest increase of antigen-specific cellular responses, which was statistically significant when compared with animals boosted with Ad26.COV2.S (p ≤ 0.026, Mann-Whitney test) (Fig. 4a and c). In naïve rhesus macaques, a single immunization with Ad26.COV2.S.529 elicited comparable WA1/2020 and Omicron S BA.1-specific T cell responses (Fig. 4a and c). In general, the profile of T cell responses induced by the different vaccine regimens was comparable between the WA1/2020 and Omicron BA.1 assays, indicating cross-reactivity of T cell responses, as supported by a high degree of conservation of T cell epitopes among all SARS-CoV-2 VOCs, including Omicron^18,35,36^. IFN-γ + CD4 + (Supplementary Fig. 4a and b) and CD8 + (Supplementary Fig. 4c and d) T cell responses measured by ICS, showed a higher percentage of antigen-specific IFN-γ producing CD8 + cells in the boosted animals compared with the non-boosted animals, while CD4 + cells appeared not to be boosted. Similar to the ELISpot data, comparable WA1/2020 and Omicron BA.1 T cell response profiles were also observed in ICS.

T cell responses were also measured by IFN-γ ELISpot in samples collected 2 weeks after BA.1 Omicron challenge, showing an increased number of IFN-γ-producing cells in all groups compared with responses measured before challenge, except for the animals boosted with the vaccine combination, that had the highest T cell response before challenge at week 6 (Fig. 4b and d). Ad26.COV2.S pre-immunized non-boosted animals had the highest T cell responses post-challenge, indicating that a robust recall of primary vaccination-derived antigen-specific memory T cells was induced by the Omicron BA.1 infection. WA1/2020 and Omicron BA.1 T cell response profiles were comparable also after challenge.

### Booster vaccination with Ad26.COV2.S.529 or the vaccine combination provided higher protection of the lower respiratory tract against SARS-CoV-2 Omicron BA.1 infection compared with Ad26.COV2.S

**Fig. 5:**
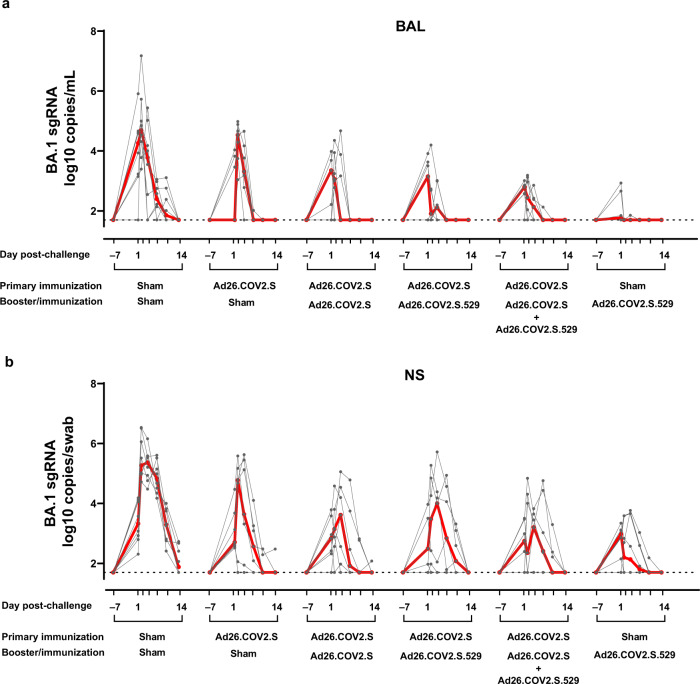
Protective efficacy against SARS-CoV-2 Omicron BA.1 inoculation after Ad26.COV2.S, Ad26.COV2.S.529, or a combination of Ad26.COV2.S and Ad26.COV2.S.529 late booster in adult rhesus macaques. Animals were challenged with 1 × 10^6^ PFU Omicron BA.1 SARS-CoV-2 administered intranasally and intratracheally 6 weeks after the booster/immunization. Viral load (sgRNA) overtime from 1 week prior to challenge up to sacrifice (day 13 or 14 post-challenge; indicated as 14 on the graphs) in **a** BAL and **b** NS expressed as log10 sgRNA copies/mL (BAL) or log10 sgRNA copies/swab (NS) from 42 NHPs. Viral loads in the individual animals are depicted with gray points and paired measurements connected with gray lines. For all panels, *n* = 7 animals per group except for the Sham-Ad26.COV2.S.529 group where *n* = 6 animals and for the Sham-Sham group where *n* = 8 animals. The median viral load per group is indicated with the red line. The dotted lines indicate the LLOD. Source data are provided as a Source Data file. BAL bronchoalveolar lavage, LLOD lower limit of detection, NHP nonhuman primate, NS nasal swab, PFU plaque-forming units, sgRNA subgenomic RNA.

At week 6 after the booster/immunization, all animals were challenged with 10^6^ plaque-forming units (PFU) SARS-CoV-2 Omicron BA.1 via the intranasal and intratracheal routes, as previously described^37^. The challenge stock was obtained from Emory University (Atlanta, GA)^38^ and was generated in VeroE6-TMPRSS2 cells. Viral loads as measure of protection, were assessed in bronchoalveolar lavage (BAL) and nasal swab (NS) samples collected pre-challenge and on day 1, 2, 4, 7, 10 and 13-14 post-challenge. Animals in the naïve sham group showed median viral load of 4.69 (range 3.40–7.18) log10 sgRNA copies/mL in BAL on day 2, which declined by day 7 to median levels of 2.40 (range 1.70–3.05) log10 sgRNA copies/mL (Fig. 5a). Previously immunized animals that did not receive a booster immunization showed median viral loads of 4.53 (range 3.04–4.89) log10 sgRNA copies/mL in BAL on day 2, which declined to median levels below the detection limit (1.70 log sgRNA copies/mL, range 1.70-2.02) by day 7, indicating that the primary vaccination applied twenty months earlier was associated with a faster control of the infection. Most boosted animals showed breakthrough infection in BAL, but viral loads were substantially lower compared with naïve sham controls, and in most animals, viral load was no longer detectable by day 4 (Fig. 5a). Peak viral load in Ad26.COV2.S, Ad26.COV2.S.529 or vaccine combination booster animals were 30-, 146- or 205-fold lower (*p* < 0.001, Tobit ANOVA z-test), respectively, compared with animals in the naïve sham group (Supplementary Fig. 5a). Peak viral load in Ad26.COV2.S.529 or vaccine combination boosted animals were 4.9- and 6.9-fold lower (*p* = 0.042 and *p* = 0.014, respectively, Tobit ANOVA z-test) compared with viral load in animals boosted with Ad26.COV2.S (Supplementary Fig. 5a). Even in animals that did not receive a booster immunization, the mean peak viral load was 7.8-fold lower (*p* = 0.035, Tobit ANOVA z-test) compared with animals in the naïve sham group (Supplementary Fig. 5a), indicating that partial protection was provided by the primary vaccination. Quantification of total viral load in BAL in the follow-up period per animal, as determined by area under the curve (AUC) analysis, showed that all boosted groups had a significantly lower total viral load compared with the naïve sham group (*p* < 0.001, Tobit ANOVA z-test). While AUC viral load was comparable between animals boosted with Ad26.COV2.S and Ad26.COV2.S.529, it was 5.6-fold lower in animals boosted with the vaccine combination compared with the Ad26.COV2.S group (*p* = 0.013, Tobit ANOVA z-test) (Supplementary Fig. 5b).

A single dose Ad26.COV2.S.529 vaccination of naïve rhesus macaques resulted in the most potent protection against Omicron BA.1 challenge, as indicated by undetectable BAL mean viral loads for almost all animals at all measured time points (Fig. 5a). These results are consistent with homologous protection previously reported in naïve rhesus macaques immunized with a single dose Ad26.COV2.S and challenged 6 or 10 weeks post-immunization with SARS-CoV-2 WA1/2020 or B.1^32,33^, respectively. Compared with the boosted animals, AUC and peak BAL viral load were significantly lower (*p* ≤ 0.022, Mann-Whitney test) in naïve animals receiving a single dose of Ad26.COV2.S.529 (Supplementary Fig. 5a and 5b).

In NS, sham controls showed high median virus levels both on day 2 post-challenge (5.27 log10 sgRNA copies/swab [range 6.54-4.72]) and day 4 post-challenge (5.37 log10 sgRNA copies/swab [range 6.16-4.48]). These levels only declined minimally by day 7 post-challenge and viral load was still detectable in half of the animals at day 14 post-challenge (Fig. 5b). Previously immunized animals that did not receive a booster immunization showed lower median viral loads of 4.78 (range 1.70–5.59) log10 sgRNA copies/swab in NS on day 2 post-challenge, which were undetectable (1.70 log10 sgRNA copies/swab [range, 1.70-2.48]) by day 10 post-challenge, indicating that the primary vaccination applied twenty months earlier was associated with a faster control of the infection, also in the upper respiratory tract. All boosted animals showed breakthrough infection in NS, but as for BAL samples, viral loads were much lower compared with sham controls and in most animals viral load was undetectable by day 7 post-challenge (Fig. 5b). Peak viral load in Ad26.COV2.S, Ad26.COV2.S.529 or vaccine combination boosted animals were similar and 70-, 70- or 60-fold lower (*p* ≤ 0.008, Tobit ANOVA z-test), respectively, compared with animals in the sham control group (Supplementary Fig. 6a). AUC viral load was significantly lower in all boosted groups compared with the sham control group (*p* ≤ 0.008, Tobit ANOVA z-test) and it was comparable among animals in the different booster groups (Supplementary Fig. 6b). Also all naïve rhesus macaques immunized with a single dose of Ad26.COV2.S.529 showed breakthrough infection in NS (Fig. 5b). Protection of this group, as measured by peak and AUC viral load, was comparable with the protection of boosted animals (Supplementary Fig. 6).

Histological analysis of lung tissue performed at the end of the challenge phase, overall confirms the protective efficacy data determined by viral load in BAL samples. We observed statistically significant levels of protection from histopathological signs of BA.1 infection conferred by the different vaccination regimens, including the non-boosted regimen, when compared with the sham control group (*p* < 0.001, Tobit ANOVA z-test). Ad26.COV2.S pre-immunized animals boosted with Ad26.COV2.S.529, the combination of the two vaccines and naïve NHPs immunized with a single dose of Ad26.COV2.S.529, showed slightly better protection against the development of lung pathology, compared with animals that did not receive a late booster immunization or that received a booster with Ad26.COV2 (Supplementary Figs. 7 and 8). Interestingly and differently from WA1/2020, Omicron BA.1 challenge also caused viral-induced inflammatory findings in the trachea, pharynx and on the nasal septum, which were comparable between vaccinated and unvaccinated animals in these tissues.

### Both humoral and cellular immune responses are correlates of protection against SARS-CoV-2 Omicron BA.1 infection

**Fig. 6:**
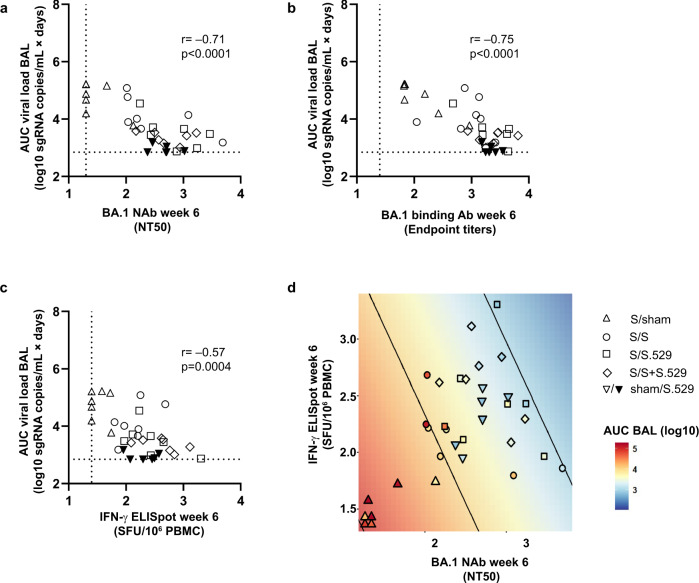
Correlates of protection analysis. Correlations of week 6 **a** Omicron BA.1 neutralizing antibodies, as measured by psVNA (NT50); **b** Omicron BA.1 binding antibodies, as measured by ELISA (endpoint titers); and **c** Omicron BA.1 T cell responses, as measured by IFN-γ ELISpot (SFU/10^6^ PBMC) with AUC viral load (sgRNA copies/mL × days) in BAL samples. The control group that underwent sham immunization and sham boosting is not included. Dotted lines indicate the LLOD for each assay. Correlations were assessed by two-sided exact Spearman rank correlation tests. **d** Regression of log10 AUC BAL sgRNA on log10 Omicron BA.1 pseudovirus neutralizing antibody titer and log10 T cell responses. In the figure, the symbols indicate the AUC BAL sgRNA of each NHP as color and their treatment group as shape against a colored background, with contours corresponding to the fitted regression model. For all 4 panels (**a**–**d**), the different symbols indicate the different immunization regimens, as indicated in the legend in panel **d** with Ad26.COV2.S indicated as “S” and Ad26.COV2.S.529 indicated as “S.529”. For all panels*, n* = 7 animals per group except for the Sham-S.529 group where *n* = 6 animals. Source data are provided as a Source Data file. Ab antibody, AUC area under the curve, BAL bronchoalveolar lavage, ELISA enzyme-linked immunosorbent assay, ELISpot enzyme-linked immunospot assay, IFN-γ interferon gamma, LLOD lower limit of detection, NAb neutralizing antibody, NHP nonhuman primate, NT50 50% neutralization titer, PBMC peripheral blood mononuclear cell, psVNA pseudovirus neutralization assay, S spike, SFU spot-forming units, sgRNA subgenomic RNA.

A correlate of protection analysis was performed to assess with data from vaccinated animals the contribution of humoral and cellular immune responses to protection of NHPs against Omicron BA.1 lower respiratory tract infection. The Omicron BA.1 neutralizing antibody titer at week 6 after the booster/immunization (time of challenge) inversely correlated with total viral load (AUC) in BAL (*p* < 0.0001, *r* = −0.71, two-sided Spearman rank-correlation test) (Fig. 6a). Similarly, Omicron BA.1 binding antibody titers inversely correlated with protection (*p* < 0.0001, *r* = −0.75, two-sided Spearman rank-correlation test) (Fig. 6b). T cell responses measured by ELISpot also inversely correlated with total viral load in BAL *(p* = 0.0004, *r* = −0,57, two-sided Spearman rank-correlation test) (Fig. 6c). These data indicate that both antibody and T cell responses may have contributed to the observed protection. In addition, in a linear regression analysis of total viral load both, log10 Omicron BA.1 pseudovirus neutralizing antibody titer (p < 0.001) and log10 T cell responses (*p* = 0.033) significantly contributed to the regression model, indicating that both parameters have complementary predictive value (Fig. 6d). Of note, the regression model did not accurately predict the protection observed in naïve animals immunized with a single dose of Ad26.COV2.S.529 (triangles down, Fig. 6a), suggesting that only the magnitude of immune responses does not explain the outcome for this group. Removing this group in an exploratory sensitivity analysis resulted in an improved prediction model, with significant contributions from both immunological parameters, pVNA and ELISpot (Supplementary Fig. 9).

## Discussion

Booster immunization with SARS-CoV-2 vaccines based on the ancestral Wuhan-Hu-1 S have shown to be very effective in preventing severe COVID-19, hospitalization, and death, including cases caused by SARS-CoV-2 Omicron variants^10,13,17,31,39^. The VOCs have shown escape from neutralizing antibody activity^8,11–15^ impacting vaccine-mediated protection against infection and moderate disease^40^. In this study, we compared the immunogenicity and efficacy of booster vaccination with Ad26.COV2.S, Ad26.COV2.S.529 (the variant vaccine encoding Omicron BA.1 spike), or the combination of the two vaccines, against SARS-CoV-2 Omicron BA.1 challenge in NHP that had been immunized twenty months earlier with Ad26.COV2.S^32^. We also included a group of naïve NHP that received a single immunization of Ad26.COV2.S.529, to assess the protection of an Omicron-based vaccine against SARS-CoV-2 Omicron BA.1 challenge in a naïve context. We observed that vaccine-matched S WA1/2020 antibody, B and T cell responses were still detectable at one and a half years post-primary immunization with Ad26.COV2.S, in line with the observed durability of Ad26.COV2.S elicited immune responses in humans, as well as immune responses elicited by Ad26-based vaccines against Zika and RSV^41–43^. Additionally, Ad26.COV2.S-elicited responses showed low levels of cross-reactivity to Omicron. Following the booster immunization, an anamnestic response resulting in a rapid and robust increase of binding and neutralizing antibody titers against both WA1/2020 and Omicron was observed independently of the booster regimen. The rapid increase in Omicron-reactive antibody responses following Ad26.COV2.S booster, may indicate that it recalled pre-existing cross-reactive memory B cells, as supported by the memory B cell analysis performed in this study, showing that most of the detected memory B cells were WA1/2020 RBD-reactive or cross-reactive to WA1/2020 and Omicron BA.1. This has also been reported for NHP and humans boosted with mRNA vaccines based on the ancestral Wuhan Hu-1 S or Omicron BA.1 S^24,25^ as well as in vaccinated people who experienced a breakthrough infection with Omicron^44–46^. The magnitude of binding and neutralizing WA1/2020, Omicron BA.1 and Omicron BA.2 antibody responses induced by the different boosters were mostly comparable, which is consistent with data reported for mRNA vaccines in NHP^24^. However, Ad26.COV2.S.529 and the vaccine combination induced 2- to 3-fold higher Omicron BA.2 neutralizing and Omicron BA.1 and BA.2 binding titers compared with Ad26.COV2.S, indicating a moderate benefit for Omicron-specific immunogenicity provided by an Omicron-matched vaccine, in line with data reported for mRNA vaccine in humans^47^. Following a booster immunization with Ad26.COV2.S, Ad26.COV2.S.529 or the vaccine combination, significantly higher protection against Omicron BA.1 infection in the lower respiratory tract was observed compared with the control group, as indicated by lower viral load readouts. Although many boosted animals showed breakthrough infection after challenge, viral load was undetectable by day 4 post-infection in most animals, while in the control group, viral load was still detectable at day 7-10 post-infection. Animals boosted with Ad26.COV2.S.529 or the combination of Ad26.COV2.S.529 and Ad26.COV2.S showed significantly lower peak sgRNA compared with animals that were boosted with Ad26.COV2.S alone. However, it remains to be shown whether a similar advantage would be observed with VOC challenge virus that has lower homology to the Spike transgene of the updated vaccine. In addition, recent data in humans showed that similar neutralizing antibody titers against all variants tested (including BA.4/BA.5) were elicited by a booster with an mRNA vaccine encoding either the ancestral SARS-CoV-2 alone or both, the ancestral SARS-CoV-2 and BA.4/BA.5^48^.

Also for the upper respiratory tract, most boosted animals showed breakthrough infection upon challenge, but as for the lower respiratory tract, viral load was significantly lower and more rapidly controlled as compared with sham controls. Viral loads in the upper respiratory tract were comparable among the boosted groups, indicating no added benefit of Ad26.COV2.S.529 in preventing upper respiratory tract infection compared with an Ad26.COV2.S-only booster. These findings may be partly related to recently reported data showing that SARS-CoV-2 Omicron BA.1 can also enter cells through the endosomal route (TMPRSS2 independent), making the upper respiratory tract, where only a low proportion of cells express both ACE2 and TMPRSS2, more susceptible to infection^49–51^.

The naïve animals immunized with a single dose of Ad26.COV2.S.529 developed Omicron BA.1 binding and neutralizing antibody responses measurable from week 2, which were comparable to those of boosted animals by week 6 post-immunization, the time of challenge. This allowed a comparison of immune responses mediated by primary Ad26.COV2.S.529 vaccination versus booster mediated recall responses. While in boosted animals most of the measured memory B cells were WA1/2020 RBD-reactive or cross-reactive to WA1/2020 and Omicron BA.1 RBD, in naïve Ad26.COV2.S.529 immunized animals, not surprisingly most of the memory B cells were Omicron-reactive or cross-reactive with WA1/2020 and Omicron BA.1. Naïve animals immunized with a single dose Ad26.COV2.S.529 showed the best lower respiratory tract protection against Omicron BA.1 challenge, with low levels of sgRNA detected only in two NHP at day 1 post-challenge. It is currently unclear why improved protection was observed in naïve animals dosed with Ad26.COV2.S.529 compared with Ad26.COV2.S pre-immunized animals, particularly since VNA responses were comparable between these groups at the time of challenge. Other factors, such as higher avidity antibody responses against omicron-specific epitopes, increased innate and/or epigenetically mediated immune responses^52^, that likely differ between naïve NHP and animals with pre-existing spike-specific immune responses, may play a role here. Naïve immunized animals also showed breakthrough infection in the upper respiratory tract, and protection of this compartment was comparable with protection in boosted animals.

Based on the observation that the booster immunization mostly recalled cross-reactive S WA1/2020 and S Omicron BA.1 B cells, we speculate that de novo induction of neutralizing antibodies targeting key new epitopes in Omicron S is impaired in boosted animals, at least shortly after vaccination, likely mediated by an imprinting effect of the primary Ad26.COV2.S vaccination. Allowing for a longer B cell affinity maturation in boosted animals, could lead to de novo generation of antibodies recognizing key new Omicron epitopes^53–55^, which can more efficiently prevent infection.

A correlate of protection analysis demonstrated that both neutralizing antibodies and T cell responses contributed to the observed protection against Omicron BA.1 infection in the lower respiratory tract. In addition, a linear regression analysis suggested that both parameters additively contribute to protection in boosted animals. The regression model did not accurately predict the protection observed in naïve animals immunized with a single dose Ad26.COV2.S.529, indicating again that only the magnitude of immune responses does not explain the outcome for this group. While neutralizing antibodies are recognized to be an important mechanism of vaccine-mediated protection against SARS-CoV-2, the role of T cells may be of particular importance for protection against antigenically different VOCs that escape neutralizing antibodies, as T cell epitopes among all SARS-CoV-2 VOCs show a high degree of conservation^18,35,36^. In line with this, we here observed comparable WA1/2020 and Omicron BA.1 specific T cell responses, irrespective of the composition of the booster vaccination. While T cells are not expected to be effective in preventing SARS-CoV-2 infection, they likely play an important role in limiting COVID-19 severity^35,56–58^. In our study we indeed showed that all vaccinated animals were significantly protected against lung damage associated with Omicron BA.1 infection compared with the sham control group. This is also in line with the effectiveness at preventing severe disease observed for the COVID-19 vaccines based on the ancestral Wuhan Hu-1 S against VOCs and with T cell-mediated protection reported for other respiratory infections^59^.

Taken together, our data in rhesus macaques suggest that an Omicron-based booster vaccine and a booster in which the original Wuhan-based and an Omicron-based vaccine are combined, can offer only moderately improved Omicron neutralizing antibody responses and protection from Omicron infection, especially of the lower respiratory tract, compared with a booster with the original Wuhan-based vaccine. It is currently unknown if this only moderate improvement is limited to the vaccine-matched Omicron VOC. Our data support real-world evidence studies on vaccine effectiveness^31,60,61^, that demonstrated that boosting with the original Wuhan S protein-based vaccines continues to provide robust protection from severe COVID-19 disease and hospitalization due to SARS-CoV-2 VOC infections.

## Methods

### Animals

The NHP study was conducted at BIOQUAL, Inc. (Rockville, MD, USA). NHPs were obtained from Charles River Laboratories (Laval, Quebec, Canada) in July 2020. The evaluations were performed in accordance with the standard operating procedures by technical staff. Animal experiment approval was provided by the Institutional Animal Care and Use Committee (IACUC) at BIOQUAL, Inc. (Rockville, MD, USA). The test facility is accredited by the American Association for Accreditation of Laboratory Animal Care (AAALAC), and animal experiments were performed in accordance with the standards of the AAALAC International’s reference resource: the eighth edition of the Guide for the Care and Use of Laboratory Animals, the Animal Welfare Act as amended, and the 2015 reprint of the Public Health Service Policy on Humane Care and Use of Laboratory Animals.

### Vaccines

Replication-incompetent E1/E3-deleted adenovirus serotype 26 (Ad26) vector-based vaccines were generated using the AdVac system^62,63^. Ad26.COV2.S encodes a SARS-COV-2 spike protein sequence based on the SARS-CoV-2 Wuhan-Hu-1 spike protein (GenBank accession number MN908947)^64^, while Ad26.COV2.S.529 encodes a SARS-COV-2 spike protein sequence based on the SARS-CoV-2 Omicron BA.1 spike protein (GISAID accession number EPI_ISL_6913991 [https://gisaid.org]). Spike proteins encoded by Ad26.COV2.S and Ad26.COV2.S.529 were stabilized in the prefusion conformation by the proline substitutions K986P and V987P and substitutions R682S and R685G, which abolish the furin cleavage site. Adenoviral vectors were tested for bioburden and endotoxin levels prior to use.

### Study design animal experiments

A total of 42 rhesus macaques (*Macaca mulatta*) of Chinese origin between 4.8 and 8.7 years of age and 4.0 and 6.9 kg were assigned to 6 experimental groups (36 females and 6 males with 4 males allocated to test group 1, and 2 males allocated to test group 6). 28 of the total 42 rhesus macaques received an initial intramuscular immunization in June 2020 consisting of either a single low dose of Ad26.COV2.S vaccine (5 × 10^10^ vp), a single high dose of Ad26.COV2.S (10 × 10^11^ vp), 2 doses of Ad26.COV2.S administered 4 weeks apart (5 × 10^10^ vp), or 2 doses of Ad26.COV2.S administered 8 weeks apart (5 × 10^10^ vp). These 28 NHP were all females due to general shortage of experimental NHP in the US and only availability of females at the start of study. Initial immunization and the results associated are described in Solforosi et al.^32^. These 28 rhesus macaques were allocated to test groups 2, 3, 4, and 5 using a randomizing stratification system based on immunization, body weight, age, and SARS-CoV-2 Wuhan spike neutralizing antibody titers and SARS-CoV-2 Wuhan spike binding antibody titers (both measured at 16 weeks after the first immunization). Eight additional rhesus macaques who had previously received an Ad26-based HIV vaccine in August 2020 were allocated to group 1. Finally, 6 rhesus macaques who had received a saline injection in June 2020 were allocated to group 6. The different vaccine regimens for this study were as follows: Group 1 and 2 (n = 8 and n = 7, respectively) were the sham control groups and received 1 saline injection at week 0. Group 3 (*n* = 7) received 1 immunization with 5 × 10^10^ vp of Ad26.COV2.S at week 0. Group 4 (n = 7) received 1 immunization with 5 × 10^10^ vp of Ad26.COV2.S.529 at week 0. Group 5 (*n* = 7) received 1 immunization with a combination of 2.5 × 10^10^ vp of Ad26.COV2.S and 2.5 × 10^10^ vp of Ad26.COV2.S.529 (for a total dose of 5 × 10^10^ vp of the bivalent combination). Group 6 (*n* = 6) received 5 × 10^10^ vp of Ad26.COV2.S.529 at week 0. All immunizations were performed via the intramuscular route in the quadriceps muscle of the left hind leg. Blood for serum and PBMC isolation was obtained as indicated in the text. Six weeks after the late booster/immunization dose, all groups were inoculated with 1 × 10^6^ PFU of SARS-CoV-2 BA.1 isolate obtained from Mehul Suthar at Emory University (Atlanta, GA, USA; GISAID accession ID: EPI_ISL_7171744 [https://gisaid.org]). The stock had a titer of 2.45 × 10^7^ PFU/mL, and the sequence was fully verified (GISAID accession ID: EPI_ISL_7171744 [https://gisaid.org]). Further information on the virus stock can be found in Edara et al.^38^. The inoculum was administered in a 2 mL volume just below the vocal cords, 1 mL intratracheally, and 1 mL intranasally (0.5 mL per nostril). After virus inoculation, nasal and trachea swabs and BAL were taken to measure viral load at day 1, day 2, day 4, day 7, day 10 post-inoculation, and on the day of sacrifice. Animals were euthanized at day 13 and 14 after virus inoculation, with the number of animals of each group evenly distributed over both days, and respiratory tract tissues were isolated for histopathology.

### ELISA for RBD WA1/2020, BA.1-, and BA.2-specific binding antibody responses to booster vaccination

IgG binding to SARS-CoV-2 spike protein was measured by ELISA using the RBD of a spike protein antigen based on the WA1/2020 SARS-CoV-2 strain (Wuhan/WIV04/2019, GISAID accession ID: EPI_ISL_402124 [https://gisaid.org/wiv04/]), the Omicron BA.1 SARS-CoV-2 strain (GISAID accession ID: EPI_ISL_7358094.2 [https://gisaid.org]), and the Omicron BA.2 SARS-CoV-2 strain (GISAID accession ID: EPI_ISL_6795834.2 [https://gisaid.org]). In brief, 96-well ELISA plates were coated with 1 µg/mL SARS-CoV-2 variant RBD protein in 1X Dulbecco’s phosphate-buffered saline (DPBS) and stored at 4 °C overnight. After a 12- to 20-h incubation overnight, plates were washed once with 200 µL 0.05% Tween 20 in DPBS in each well and blocked with 350 µL casein block/well for 2 to 3 h Serum from infected animals was heat inactivated for ≥30 min at 56 °C prior to plating. Serum was plated in a 3-fold serial dilution starting at 1:50. The plates were covered and incubated for 1 h and washed 3 times with 275 µL DPBS-Tween. Anti-macaque IgG horseradish peroxidase (HRP) (NIH Non-human Primate Reagent Resource, Cat# 1b3-HRP; 0320K235 / 070920SC) was diluted in casein block at 1 μg/mL, added to the plate, and incubated in the dark for an hour. After incubation, the plates were washed 3 times with 275 µL DPBS-Tween. After 3 washes, 100 µL of SeraCare (Milford, MA, USA) KPL tetramethylbenzidine (TMB) SureBlue Start solution were added to each well, and incubated for 9 m and 30 s. The development was halted by adding 100 µL SeraCare KPL TMB Stop solution. Plates were read at 450 nm using the VersaMax or Omega microplate reader. The raw optical density (OD) values were used to calculate a curve fit using a sigmoidal, 4-parameter logistic fit model (GraphPad Prism). To quantify ELISA endpoint titers, the interpolation function was used to calculate the dilution at which the OD value would be equal to a value of 0.2.

### psVNA for S WA1/2020, BA.1-, and BA.2-specific neutralizing antibody responses to booster vaccination

For assessment of the immunogenicity elicited by a late booster, SARS-CoV-2 spike neutralizing antibody titers were measured by psVNA. The SARS-CoV-2 pseudoviruses expressing a luciferase reporter gene were generated as described previously^65^. Briefly, 10 µg packaging construct psPAX2 (Cat#11348, AIDS Reagent), 10 µg luciferase reporter plasmid pLenti CMV Puro LUC (Cat#17447, Addgene) and 5 µg S protein expressing pcDNA3.1-SARS CoV2.SΔCT were co-transfected into 5 × 10^6^ HEK293T cells (ATCC) with lipofectamine 2,000 (Sigma). Pseudoviruses of SARS-CoV-2 variants were generated by using the WA1/2020 strain (Wuhan/WIV04/2019, GISAID accession ID: EPI_ISL_402124 [https://gisaid.org/wiv04/]), BA.1 (Omicron, GISAID accession ID: EPI_ISL_7358094.2 [https://gisaid.org]), and BA.2 (GISAID accession ID: EPI_ISL_6795834.2 [https://gisaid.org]).

Six hours post-transfection, the supernatants were replaced with fresh DMEM (plus 5% FBS). The supernatants containing the pseudotype viruses were collected 48 hs post-transfection; pseudotype viruses were purified by filtration with a 0.45 µm filter. To determine the neutralization activity of the antisera from vaccinated animals, stably transfected HEK293T-hACE2 cells were seeded in 96-well tissue culture plates at a density of 1.75 × 10^4^ cells/well overnight. Six 3-fold serial dilutions of heat-inactivated serum samples were prepared and mixed with 50 µL of pseudovirus. The mixture was incubated at 37 °C for 1 h before it was added to HEK293T-hACE2 cells. 48 h after infection, cells were lysed in Steady-Glo Luciferase Assay (Promega) according to the manufacturer’s instructions. SARS-CoV-2 neutralization titers were defined as the interpolated sample dilution at which a 50% reduction in relative light units (RLU) was observed relative to the average of the virus control wells.

### ECLA

ECLA plates (Meso Scale Discovery [MSD] SARS-CoV-2 IgG, Panels 22, 23) were designed and produced for multiplex binding assays with up to 10 antigen spots per well, including either spike or RBD proteins from multiple SARS-CoV-2 variants^34^. The plates were blocked with 50 μL of Blocker A (1% BSA in distilled water) solution for ≥30 min at room temperature shaking on a digital microplate shaker. During blocking, the serum was diluted to 1:5,000 or 1:50,000 in Diluent 100. The calibrator curve was prepared by diluting the calibrator mixture from MSD 1:10 in Diluent 100 and then preparing a 7-step 4-fold dilution series plus a blank containing only Diluent 100. The plates were then washed 3 times with 150 μL of wash buffer (0.5% Tween in 1X PBS) and blotted dry, and 50 μL of the samples and calibration curve were added in duplicate to the plates and set to shake at room temperature for ≥2 h. The plates were washed 3 times and 50 μL of SULFO-TAG anti-human IgG detection antibody diluted to 1X in Diluent 100 was added to each well and incubated shaking at room temperature for ≥1 h. Plates were then washed 3 times, and 150 μL of MSD GOLD Read Buffer B was added to each well; the plates were read immediately after on a MESO QuickPlex SQ 120 machine. MSD titers for each sample was reported as RLU, which were calculated as sample RLU minus blank RLU and then fit using a logarithmic fit to the standard curve. The upper limit of detection was defined as 2 × 10^6^ RLU for each assay; samples with a signal that exceeded this value at 1:5,000 serum dilutions were run again at 1:50,000 and the fitted RLU was multiplied by 10 before reporting. The LLOD was defined as 1 RLU, and an RLU value of 100 was defined to be positive for each assay.

### B cell profiling

Fresh PBMCs were stained with Aqua LIVE/DEAD dye for 20 min and washed with 2% FBS/DPBS buffer; cells were suspended in 2% FBS/DPBS buffer with Fc Block (BD Biosciences) for 10 min, followed by staining with monoclonal antibodies (BD Biosciences) against CD45 (clone D058-1283, BUV805, BD Biosciences, Cat#742055), CD3 (clone SP34.2, APC-Cy7, BD Biosciences, Cat#557757), CD7 (clone M-T701, Alexa700, BD Biosciences, Cat#561603), CD123 (clone 6H6, Alexa700, Biolegend, Cat#306040), CD11c (clone 3.9, Alexa700, Biolegend, Cat#301648), CD20 (clone 2H7, PE-Cy5, BD Biosciences, Cat#555624), IgG (clone G18-145, BUV737, BD Biosciences, Cat#612819), IgM (clone G20-127, BUV395, BD Biosciences, Cat#563903), CD27 (clone M-T271, BUV563, BD Biosciences, Cat#741366), CD21 (clone B-ly4, BV605, BD Biosciences, Cat#740395), CD14 (clone M5E2, Alexa700, Biolegend, Cat#301832), and staining with SARS-CoV-2 antigens, including WA1/2020 RBD Biotin (Sino Biological, Cat#40592-V08B-B) labelled with BV650 streptavidin (BD Bioscience, Cat#563855), WA1/2020 RBD (Sino Biological, Cat#40592-V08B) labelled with FITC Conjugation Kit (Fast) - Lightning-Link® (Abcam, Cat# ab188285), SARS-CoV-2 B.1.1.529 (Omicron BA.1) RBD (Sino Biological, Cat#40592-V08H121) labelled with DyLight® 405 Conjugation Kit (Fast) - Lightning-Link® (Abcam, Cat # ab201798) or APC by Alexa Fluor® 647 Conjugation Kit (Fast) - Lightning-Link® (Abcam, Cat # ab269823). Staining was done at 4 °C for 30 min. After staining, cells were washed twice with 2% FBS/DPBS buffer, followed by incubation with BV650 streptavidin (BD Pharmingen, Cat#563855) for 10 min, and then washed twice with 2% FBS/DPBS buffer. After staining, cells were washed and fixed by 2% paraformaldehyde. All data were acquired on a BD FACSymphony™ flow cytometer. Subsequent analyses were performed using FlowJo software (BD Bioscience, v10). For analyses, in singlet gate, dead cells were excluded by Aqua dye and CD45 was used as a positive inclusion gate for all leukocytes. Within class-switched B cell populations, gated as CD20 + IgG+ IgM-CD3-CD14-CD11c-CD123-CD7-, SARS-CoV-2 WA1/2020 RBD-reactive B cells were identified as double positive for SARS-CoV-2 (WA1/2020) RBD labeled with different fluorescent probes, and SARS-CoV-2 Omicron BA.1 RBD-reactive B cells were identified as double positive for SARS-CoV-2 (BA.1) RBD proteins labeled with different fluorescent probes. Within the antigen-reactive B cells, memory B cells were identified as CD21 + and CD27 + . The gaining strategy is shown in Supplementary Fig. 10.

### ELISpot

IFN-γ ELISpot was performed on freshly isolated PBMC. In brief, ELISpot plates were coated with IFN-γ coating antibody (rabbit polyclonal anti-human IFN-γ, U-Cytech, Cat# CT243) at 5 µg/well overnight at 4 °C. Plates were washed with DPBS containing 0.25% Tween 20 and blocked with R10 media (500 mL RPM1460 with 55 mL PFBS and 5.5 mL of 100X penicillin-streptomycin) for 1 to 4 h at 37 °C. The peptides (1 µg/well) and cells (2 × 10^5^/well) were added to the plate and incubated for 18 to 24 h at 37 °C. All steps following this incubation were performed at room temperature. The plates were washed with Coulter buffer and incubated for 2 h with biotinylated antibody (1 µg/mL). The plates were washed a second time and incubated for 2 h with streptavidin-AP antibody (2 µg/mL). The final wash was followed by the addition of warmed and filtered nitro blue tetrazolium chloride/5-bromo-4-chloro 3 ‘indolyl phosphate p-toluidine salt (BCIP/NBT chromogen) substrate solution for 7 min. The chromogen was discarded, and the plates were washed with water and dried in a dim place for 24 h. Plates were scanned and counted. Results are expressed with a background subtraction based on the medium control wells prior to calculating the sum of stimulations and imputing values below or at LLOD to 25 spots/10^6^ cells per peptide pool.

### ICS

CD4 + and CD8 + T-cell responses were quantitated by pooled peptide-stimulated ICS assays. Peptide pools were 16 amino acid peptides overlapping by 11 amino acids spanning the SARS-CoV-2 WA1/2020 or BA.1 (Omicron; GISAID accession ID: EPI_ISL_7358094.2 [https://gisaid.org]) spike proteins (21^st^ Century Biochemicals). 10^6^ PBMCs were re-suspended in 100 µL of R10 media supplemented with monoclonal antibodies against CD49d (1 µg/mL) and CD28 (1 µg/mL). Each sample was assessed with mock (100 µL of R10 plus 0.5% DMSO; background control), peptides (2 µg/mL), and/or 10 pg/mL phorbol myristate acetate and 1 µg/mL ionomycin (Sigma-Aldrich; 100 µL; positive control) and incubated at 37 °C for 1 hr. After incubation, 0.25 µL of GolgiStop and 0.25 µL of GolgiPlug in 50 µL of R10 were added to each well and incubated at 37 °C for 8 h and then held at 4 °C overnight. The next day, the cells were washed twice with DPBS, stained with Aqua LIVE/DEAD™ dye for 10 min, and stained with predetermined titers of monoclonal antibodies against CD279 (clone EH12.1, BB700, BD Biosciences, Cat#566460), CD38 (clone OKT10, PE, NIH NHP Reagent Resource RRID: AB_2819278), CD28 (clone 28.2, PE CY5, BD Biosciences, Cat#555730), CD4 (clone L200, BV510, BD Biosciences, Cat#563094), CD95 (clone DX2, BUV737, BD Biosciences, Cat#612790), CD8 (clone SK1, BUV805, BD Biosciences, Cat#612889) for 30 min. Cells were then washed twice with 2% FBS/DPBS buffer and incubated for 15 min with 200 µL of BD Cytofix/Cytoperm™ Fixation/Permeabilization solution. Cells were washed twice with 1X Perm/Wash buffer (BD Perm/Wash™ Buffer 10X in the Cytofix/Cytoperm Fixation/Permeabilization kit diluted with Milli-Q water and passed through 0.22 µm filter) and stained intracellularly with monoclonal antibodies Ki67 (clone B56, A488, BD Biosciences, Cat#561165), CD69 (clone TP1.55.3, ECD, Beckman Coulter, Cat#6607110), IL10 (clone JES3-9D7, PE CY7, Biolegend, Cat#501420), IL13 (clone JES10-5A2, BV421, BD Biosciences, Cat#563580), TNF-α (clone Mab11, BV650, BD Biosciences, Cat#563418), IL4 (clone MP4-25D2, BV711, BD Biosciences, Cat#564112), IFN-γ (clone B27; BUV395, BD Biosciences, Cat#563563), IL2 (clone MQ1-17H12, APC, BD Biosciences, Cat#554567), CD45 (clone D058-1283, BUV615, BD Biosciences, Cat#751117), CD3 (clone SP34.2, Alexa 700, BD Biosciences, Cat#557917) (BD). for 30 min. Cells were washed twice with 1X Perm/Wash buffer and fixed with 250 µL of freshly prepared 1.5% formaldehyde. Fixed cells were transferred to a 96-well round-bottom plate and analyzed by the BD FACSymphony™ system. Data were analyzed using FlowJo v10.

### RNA isolation and BA.1 SARS-CoV-2 subgenomic mRNA assay

Subgenomic reverse transcriptase–polymerase chain reaction (RT-PCR) assay SARS-CoV-2 E gene sgRNA was assessed by RT-PCR using primers and probes as previously described^66^. A standard was generated by synthesizing a gene fragment of the subgenomic E gene. The gene fragment was subsequently cloned into a pcDNA3.1+ expression plasmid using restriction site cloning (Integrated DNA Technologies). The insert was transcribed in vitro to RNA using the AmpliCap-Max T7 High Yield Message Maker Kit (CELLSCRIPT). Log dilutions of the standard were prepared for RT-PCR assays ranging from 1 × 10^10^ copies to 1 × 10^–1^ copies. Viral loads were assessed in BAL and NS samples collected pre-challenge and on days 1, 2, 4, 7, 10, and 13 to 14 postchallenge. RNA extraction was performed on a QIAcube HT using the IndiSpin QIAcube HT Pathogen Kit according to manufacturer’s specifications (QIAGEN). The standard dilutions and extracted RNA samples were reverse transcribed using SuperScript VILO Master Mix (Invitrogen) following the cycling conditions described by the manufacturer. A TaqMan custom gene expression assay (Thermo Fisher Scientific) was designed using the sequences targeting the E gene sgRNA. The sequences for the custom assay were as follows: forward primer, sgLeadCoV2. Fwd: CGATCTCTTGTAGATCTGTTCTC, E_Sarbeco_R: ATATTGCAGCAGTACGCACACA, E_Sarbeco_P1 (probe): VIC-ACACTAGCCATCCTTACTGCGCTTCG-MGBNFQ. Reactions were carried out in duplicate for samples and standards on the QuantStudio 6 and 7 Flex Real-Time PCR Systems (Applied Biosystems) with the thermal cycling conditions: initial denaturation at 95 °C for 20 s, 45 cycles of 95 °C for 1 s and 60 °C for 20 seconds. Standard curves were used to calculate subgenomic RNA copies per mL or per swab. The quantitative assay sensitivity was determined as 50 copies per mL or per swab.

### Lung gross pathology and histopathology

At the end of the follow-up period all animals were necropsied by opening the thoracic and abdominal cavities and all major organs were examined. The extent of pulmonary consolidation was assessed based on visual estimation of the percentage of affected lung tissue. Nasal mucosa, pharynx, trachea, bronchi and all lung lobes were collected for histopathological examination and analysis by immunohistochemistry. All tissues were immersed in 10% neutral-buffered formalin for fixation, paraffin embedded and stained with hematoxylin and eosin (H&E) for histopathological evaluation. The histopathology evaluation of H&E-stained tissue sections was conducted by a board-certified pathologist with expertise/experience in respiratory tract pathology in NHPs^67–69^ and was performed digitally using whole slide images (WSI) scanned by an Aperio scanner (Leica Biosystems) at X40, and viewed using Halo Link browser (Indica Labs). Lung histopathology scores were obtained by the evaluation of 7 lung lobes from each animal, and the assignment of a severity grade to a predetermined list of findings. The slides were evaluated with no knowledge of the group (blind evaluation), scores were tallied for each lung, and then for each animal. Groups scores were generated once the animal identity was known.

### Statistical analysis

The statistical analysis was performed using GraphPad Prism 9.4.1, Statistical Analysis System (SAS) 9.4 and R.4.0.2.

#### ELISA, psVNA, viral load, and histopathology data

Peak and AUC viral load and assay titers are log transformed; histology score is not. An ANOVA is applied with immunization regimen as a factor. An ANOVA is performed with post hoc t-tests for the primary comparisons of the immunization groups with the unimmunized group with a 5-fold Bonferroni adjustment for multiple comparisons. Additionally, an ANOVA is performed for the secondary pairwise comparisons of all immunization regimens except for the comparison of COV2.S/sham with sham/COV2.S.529. If censoring at the LLOQ occurs, then a Tobit ANOVA with post hoc z-tests is used instead of ANOVA and t-tests. In case of ≥50% censoring in a group, the non-parametric Mann-Whitney U-test is used for its comparisons. A statistical significance level of 5% is used.

#### Correlation analysis

Correlation coefficients between viral load (AUC and peak) and neutralizing antibody titers, binding antibody concentrations, or IFN-γ ELISpot results were calculated using two-sided Spearman rank correlation.

#### Regression analysis

The viral load, as measured by sgRNA in BAL samples, is summarized as AUC over the two-week period after challenge. Linear regression of the log-transformed AUC is calculated on log- transformed VNA and ELISpot as covariables.

### Reporting summary

Further information on research design is available in the Nature Portfolio Reporting Summary linked to this article.

## Supplementary information

Supplementary Information

Reporting Summary

## Data Availability

All data generated or analyzed during this study are included in this published article and its supplementary information files. Source data are provided with this paper.

## Source data

Source Data
