# Supplementary material for: Booster with Ad26.COV2.S or Omicron-adapted vaccine enhanced immunity and efficacy against SARS-CoV-2 Omicron in macaques
Source: Nat Commun. 2023 Apr 7;14:1944. doi: 10.1038/s41467-023-37715-2 (PMC10080532; doi:10.1038/s41467-023-37715-2)
Supplement: Supplementary file 1 — Supplementary Information [file 41467_2023_37715_MOESM1_ESM.pdf]

Supplementary Information

Booster with Ad26.COV2.S or Omicron-adapted vaccine enhanced immunity and efficacy against SARS-CoV-2 Omicron in macaques

Laura Solforosi[1#]*, Lea M.M. Costes[1#], Jeroen T.B.M. Tolboom[1], Katherine McMahan[2], Tochi Anioke[2], David Hope[2], Tetyana Murdza[2], Michaela Sciacca[2], Emily Bouffard[2], Julia Barrett[2], Cindy Wu[2], Nicole Hachmann[2], Jessica Miller[2], Jingyou Yu[2], Xuan He[2], Catherine Jacob-Dolan[2], Sietske K. Rosendahl Huber[1], Liesbeth Dekking[1], Ronnie Chamanza[3], Ying Choi[1], Karin Feddes-de Boer[1], Dan H. Barouch[2,4,5,6], Hanneke Schuitemaker[1], Roland C. Zahn[1], Frank Wegmann[1]*

[1]Janssen Vaccines and Prevention B.V., Leiden, Netherlands; [2]Center for Virology and Vaccine Research, Beth Israel Deaconess Medical Center, Harvard Medical School, Boston, MA, USA; [3]Non-Clinical Safety Toxicology/Pathology, Janssen Research and Development, Beerse, Belgium; [4]Ragon Institute of MGH, MIT and Harvard, Cambridge, MA, USA; [5]Harvard Medical School, Boston, MA, USA; [6]Massachusetts Consortium on Pathogen Readiness, Boston, MA, USA.

#These authors contributed equally

*Corresponding authors: lsolforo@its.jnj.com, fwegmann@its.jnj.com

Supplementary Tables

Supplementary Table 1

Related to Fig. 3	Comparison	p value	Significance symbol
a	S - sham to sham - sham	0.035	#
a	S - S to sham - sham	0.001	###
a	S - S.529 to sham - sham	0.001	###
a	S - S+S.529 to sham - sham	0.001	###
a	sham - S.529 to sham - sham	0.015	#
a	S - S to S - sham	0.000	***
a	S - S.529 to S - sham	0.008	**
a	S - S+S.529 to S - sham	0.001	***
a	S - S to sham - S.529	0.019	*
b	S - S to sham - sham	0.007	##
b	S - S.529 to sham - sham	0.035	#
b	S - S+S.529 to sham - sham	0.035	#
b	sham - S.529 to sham - sham	0.015	#
b	S - S to S - sham	0.005	##
b	S - S.529 to S - sham	0.021	#

b	S - S+S.529 to S - sham	0.021	#
b	S - S to sham - S.529	0.009	**
b	S - S.529 to sham - S.529	0.005	**
b	S - S+S.529 to sham - S.529	0.004	**

Supplementary Table 1. Comparison for RBD-specific memory B-cell responses following vaccination. Comparisons between the sham-immunized group and all other groups with 5-fold Bonferroni adjustment as well as pairwise comparisons between all groups except the control sham-immunized group were made with a 2-sided t-test or exact Mann-Whitney U-test. T-test: *p<0.05; **p<0.01; ***p<0.001. Mann-Whitney U-test: $^{\#}$p<0.05; $^{\#\#}$p<0.01; $^{\#\#\#}$p<0.001. Non-significant comparisons are not indicated in the table. RBD, receptor-binding domain; S, spike.

Supplementary Table 2

Related to Fig. 4	Comparison	p value	Significance symbol
a	S - sham to sham - sham	0.035	#
a	S - S to sham - sham	0.001	###
a	S - S.529 to sham - sham	0.001	###
a	S - S+S.529 to sham - sham	0.001	###
a	sham - S.529 to sham - sham	0.002	##
a	S - S to S - sham	0.001	###
a	S - S.529 to S - sham	0.001	###
a	S - S+S.529 to S - sham	0.001	###
a	S - S to S - S+S.529	0.002	##
a	S - S+S.529 to sham - S.529	0.012	#
b	S - sham to sham - sham	0.002	##
b	S - S to sham - sham	0.019	#
b	S - S+S.529 to sham - sham	0.011	#
b	sham - S.529 to sham - sham	0.023	#
b	S - S to S - sham	0.007	##
b	S - S.529 to S - sham	0.007	##
b	S - S+S.529 to S - sham	0.017	#
c	S - S to sham - sham	0.001	###
c	S - S.529 to sham - sham	0.001	###
c	S - S+S.529 to sham - sham	0.001	###
c	sham - S.529 to sham - sham	0.002	##
c	S - S to S - sham	0.001	###
c	S - S.529 to S - sham	0.001	###
c	S - S+S.529 to S - sham	0.001	###
c	S - S to S - S+S.529	0.026	#
d	S - sham to sham - sham	0.002	##
d	S - S to sham - sham	0.012	#
d	S - S.529 to sham - sham	0.012	#
d	S - S+S.529 to sham - sham	0.011	#
d	sham - S.529 to sham - sham	0.023	#
d	S - S+S.529 to S - sham	0.026	#

Supplementary Table 2. SARS-CoV-2–specific cellular immune responses (IFN-γ ELISpot) after Ad26.COV2.S, Ad26.COV2.S.529, or a combination of Ad26.COV2.S and Ad26.COV2.S.529 late booster in adult rhesus macaques. Comparisons between the sham-immunized group and all other groups with 5-fold Bonferroni adjustment as well as pairwise comparisons between groups except the control sham-immunized group were made with a 2-sided z-test, t-test, or exact Mann-Whitney U-test. Mann-Whitney U-test: $^{\#}p<0.05$; $^{\#\#}p<0.01$; $^{\#\#\#}p<0.001$. Non-significant comparisons are not indicated in the table. S, spike.

Supplementary Table 3

Related to Supplementary Fig. 4	Comparison	p value	Significance symbol
a	S - sham to sham - sham	0.001	###
a	S - S to sham - sham	0.001	###
a	S - S.529 to sham - sham	0.007	##
a	S - S+S.529 to sham - sham	0.001	###
a	S - S to sham - S.529	0.005	##
a	S - S+S.529 to sham - S.529	0.008	##
b	S - sham to sham - sham	0.007	##
b	S - S to sham - sham	0.001	###
b	S - S.529 to sham - sham	0.007	##
b	S - S+S.529 to sham - sham	0.007	##
b	S - S to sham - S.529	0.013	#
c	S - S to sham - sham	0.001	###
c	S - S.529 to sham - sham	0.001	###
c	S - S+S.529 to sham - sham	0.007	##
c	S - S to S - sham	0.001	***
c	S - S.529 to S - sham	0.045	*
d	S - S to sham - sham	0.001	###
d	S - S.529 to sham - sham	0.007	##
d	S - S+S.529 to sham - sham	0.001	###
d	sham - S.529 to sham - sham	0.015	#
d	S - S to S - sham	0.001	##
d	S - S.529 to S - sham	0.018	#
d	S - S+S.529 to S - sham	0.001	##

Supplementary Table 3. Comparison SARS-CoV-2–specific cellular immune responses (ICS) after Ad26.COV2.S, Ad26.COV2.S.529, or a combination of Ad26.COV2.S and Ad26.COV2.S.529 late booster in adult rhesus macaques. Comparisons between the sham-immunized group and all other groups with 5-fold Bonferroni adjustment as well as pairwise comparisons between all groups except the control sham-immunized group were made with a 2-sided t-test or exact Mann-Whitney U-test. T-test: *p<0.05; ***p<0.001. Mann-Whitney U-test: $^{\#}$p<0.05; $^{\#\#}$p<0.01; $^{\#\#\#}$p<0.001. Non-significant comparisons are not indicated in the table. S, spike.

Supplementary Table 4

Related to Supplementary Fig. 5	Comparison	p value	Significance symbol
a	S - sham to sham - sham	0.035	*
a	S - S to sham - sham	0.000	***
a	S - S.529 to sham - sham	0.000	***
a	S - S+S.529 to sham - sham	0.000	***
a	Sham - S.529 to sham - sham	0.003	##
a	S - S to sham - S.529	0.002	##
a	S - S.529 to sham - S.529	0.013	#
a	S - S+S.529 to sham - S.529	0.013	#
a	S - sham to S - S.529	0.000	***
a	S - sham to S - S+S.529	0.000	***
a	S - S to S - S+S.529	0.014	*
a	S - S to S - S.529	0.042	*
b	S - sham to sham - sham	0.034	*
b	S - S to sham - sham	0.000	***
b	S - S.529 to sham - sham	0.000	***
b	S - S+S.529 to sham - sham	0.000	***
b	Sham - S.529 to sham - sham	0.003	##
b	S - sham to S - S	0.039	*
b	S - sham to S - S.529	0.000	***
b	S - sham to S - S+S.529	0.000	***
b	S - S to S - S+S.529	0.013	*
b	S - S to sham - S.529	0.001	##
b	Sham - S.529 to S - S.529	0.022	#
b	Sham - S.529 to S - S+S.529	0.008	##

Supplementary Table 4. Comparison of protective efficacy of the lower respiratory tract against SARS-CoV-2 Omicron BA.1 inoculation after Ad26.COV2.S, Ad26.COV2.S.529, or a combination of Ad26.COV2.S and Ad26.COV2.S.529 late booster in adult rhesus macaques. Comparisons between the sham-immunized group and all other groups with 5-fold Bonferroni adjustment as well as pairwise comparisons between all groups except the control sham-immunized group were made with a 2-sided t-test or exact Mann-Whitney U-test. T-test: *p<0.05; ***p<0.001. Mann-Whitney U-test: $^{\#}p<0.05$; $^{\#\#}p<0.01$. Non-significant comparisons are not indicated in the table. S, spike.

Supplementary Table 5

Related to Supplementary Fig. 6	Comparison	p value	Significance symbol
a	S - S to sham - sham	0.006	**
a	S - S.529 to sham - sham	0.006	**
a	S - S+S.529 to sham - sham	0.008	**
a	Sham - S.529 to sham - sham	0.000	***
b	S - S to sham - sham	0.003	**
b	S - S.529 to sham - sham	0.008	**
b	S - S+S.529 to sham - sham	0.003	**
b	Sham - S.529 to sham - sham	0.000	***

Supplementary Table 5. Comparisons of protective efficacy of the upper respiratory tract against SARS-CoV-2 Omicron BA.1 inoculation after Ad26.COV2.S, Ad26.COV2.S.529, or a combination of Ad26.COV2.S and Ad26.COV2.S.529 late booster in adult rhesus macaques. Comparisons between the sham-immunized group and all other groups with 5-fold Bonferroni adjustment as well as pairwise comparisons between all groups except the control sham-immunized group were made with a 2-sided t-test or exact Mann-Whitney U-test. T-test: **p<0.01; ***p<0.001. Non-significant comparisons are not indicated in the table. S, spike.

Supplementary Table 6

Related to Supplementary Fig. 7	Comparison	p value	Significance symbol
	S - sham to sham - sham	0.000	***
	S - S to sham - sham	0.000	***
	S - S.529 to sham - sham	0.000	***
	S - S+S.529 to sham - sham	0.000	***
	Sham - S.529 to sham - sham	0.000	***
	S - S to sham - S.529	0.005	**
	S - sham to S - S.529	0.000	***
	S - sham to S - S+S.529	0.002	**
	S - S to S - S+S.529	0.013	*
	S - S to S - S.529	0.003	**

Supplementary Table 6. Comparisons of lung histopathology score after SARS-CoV-2 Omicron BA.1 inoculation after Ad26.COV2.S, Ad26.COV2.S.529, or a combination of Ad26.COV2.S and Ad26.COV2.S.529 late booster in adult rhesus macaques. Comparisons between the sham-immunized group and all other groups with 5-fold Bonferroni adjustment as well as pairwise comparisons between all groups except the control sham-immunized group were made with a 2-sided t-test or exact Mann-Whitney U-test. T-test: **p<0.01; ***p<0.001. Non-significant comparisons are not indicated in the table. S, spike.

Supplementary Figures

Supplementary Fig. 1

a

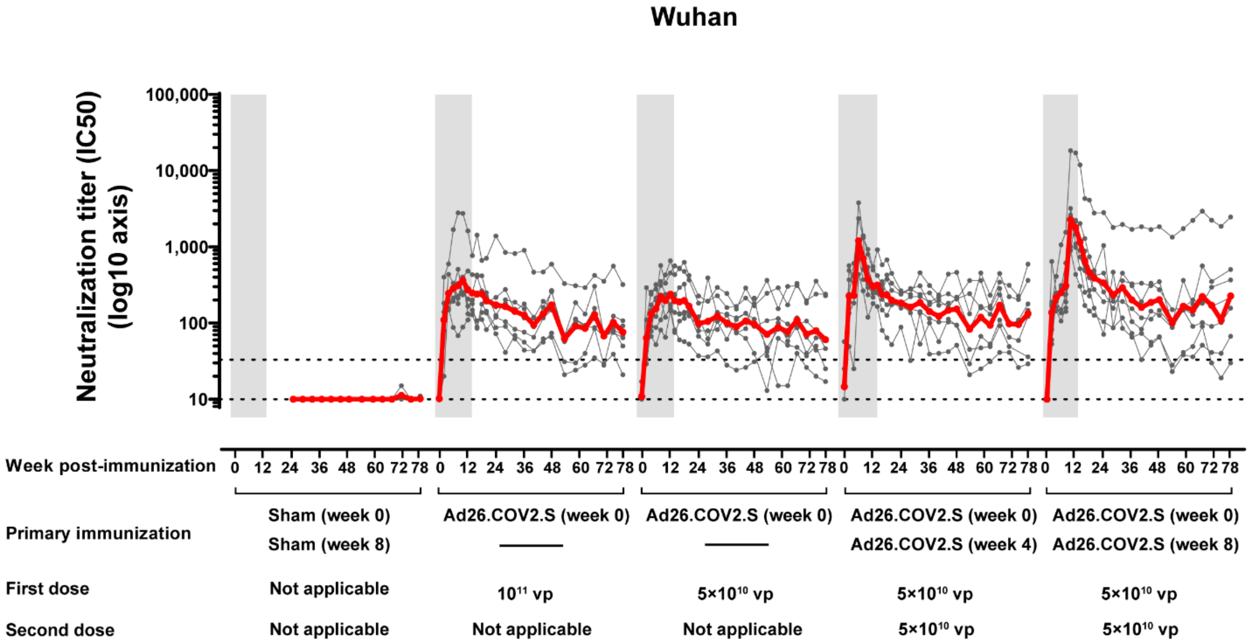

b

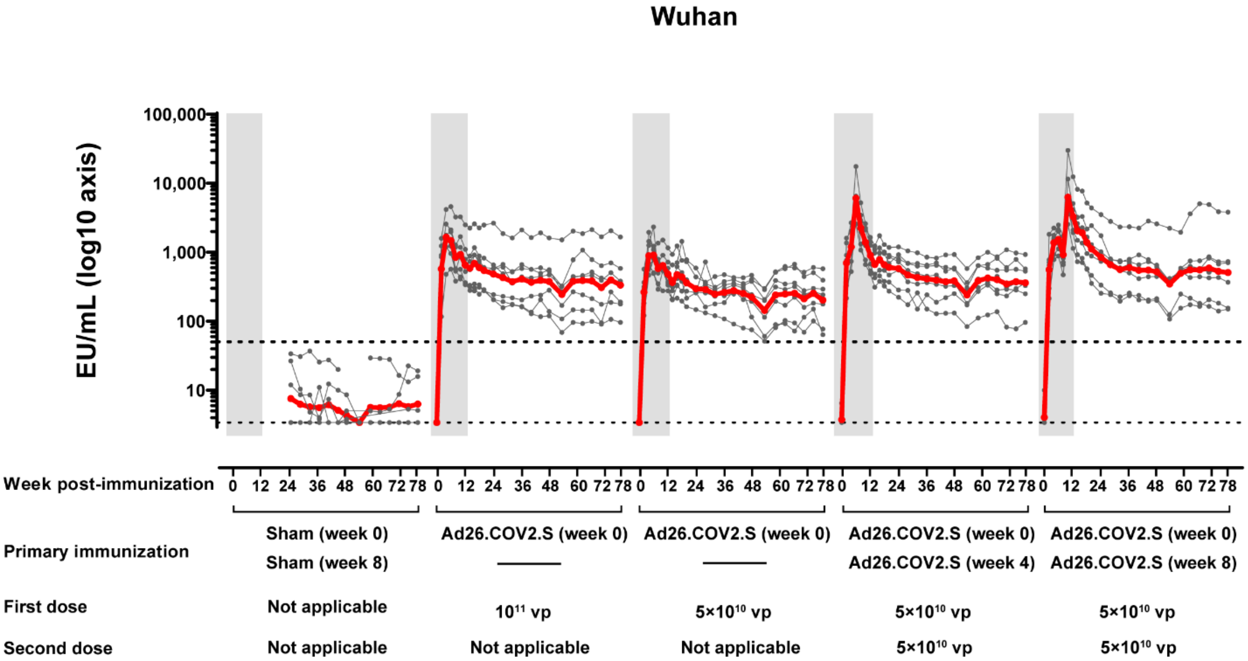

Supplementary Fig. 1. Kinetics of Wuhan S–specific neutralizing and binding antibody response after the primary vaccination with single- or 2-dose Ad26.COV2.S regimens. a SARS-CoV-2 spike protein neutralizing antibody titers were measured over time in NHP serum samples (36 NHPs and 25 time points; 106 data points not available) with a psVNA qualified for human samples, using pseudotyped virus particles made from a modified vesicular stomatitis virus (VSVΔG) backbone and bearing the S glycoprotein of SARS-CoV-2 Wuhan-Hu-1. Neutralizing antibody responses were expressed as the reciprocal of the sample dilution where 50% neutralization was achieved (IC50). Antibody levels in the individual animals are depicted with gray points and paired measurements connected with gray lines. The GMT of neutralizing antibody responses per group is indicated with the red line. The dotted lines indicate the LLOD and LLOQ generated from the qualification of the assay for human serum samples. Data in the gray area were already reported in Solforosi et al, 2021.[28] **b** SARS-CoV-2 spike protein–binding antibody concentrations were measured over time in NHP serum samples (36 NHPs and 25 time points; 116 data points not available) with an ELISA qualified for human samples, using a trimeric, soluble, stabilized spike protein produced in mammalian cells as coating antigen. Antibody levels in the individual animals are depicted with gray points and paired measurements connected with gray lines. The GMT of binding antibody responses per group is indicated with the red line. The dotted lines indicate the LLOD and LLOQ. Data in the gray area were already reported in Solforosi et al, 2021.[1] For all panels, n=7 animals per group except for the Sham-Sham group where n=7 animals. Source data are provided as a Source Data file. ELISA, enzyme-linked immunosorbent assay; EU, enzyme-linked immunosorbent assay units; GMT, geometric mean titer; IC50, 50% neutralization antibody titer; LLOD, lower limit of detection; LLOQ, lower limit of quantification; NHP, nonhuman primate; psVNA, pseudovirus neutralization assay; S, spike; vp, viral particles.

Supplementary Fig. 2

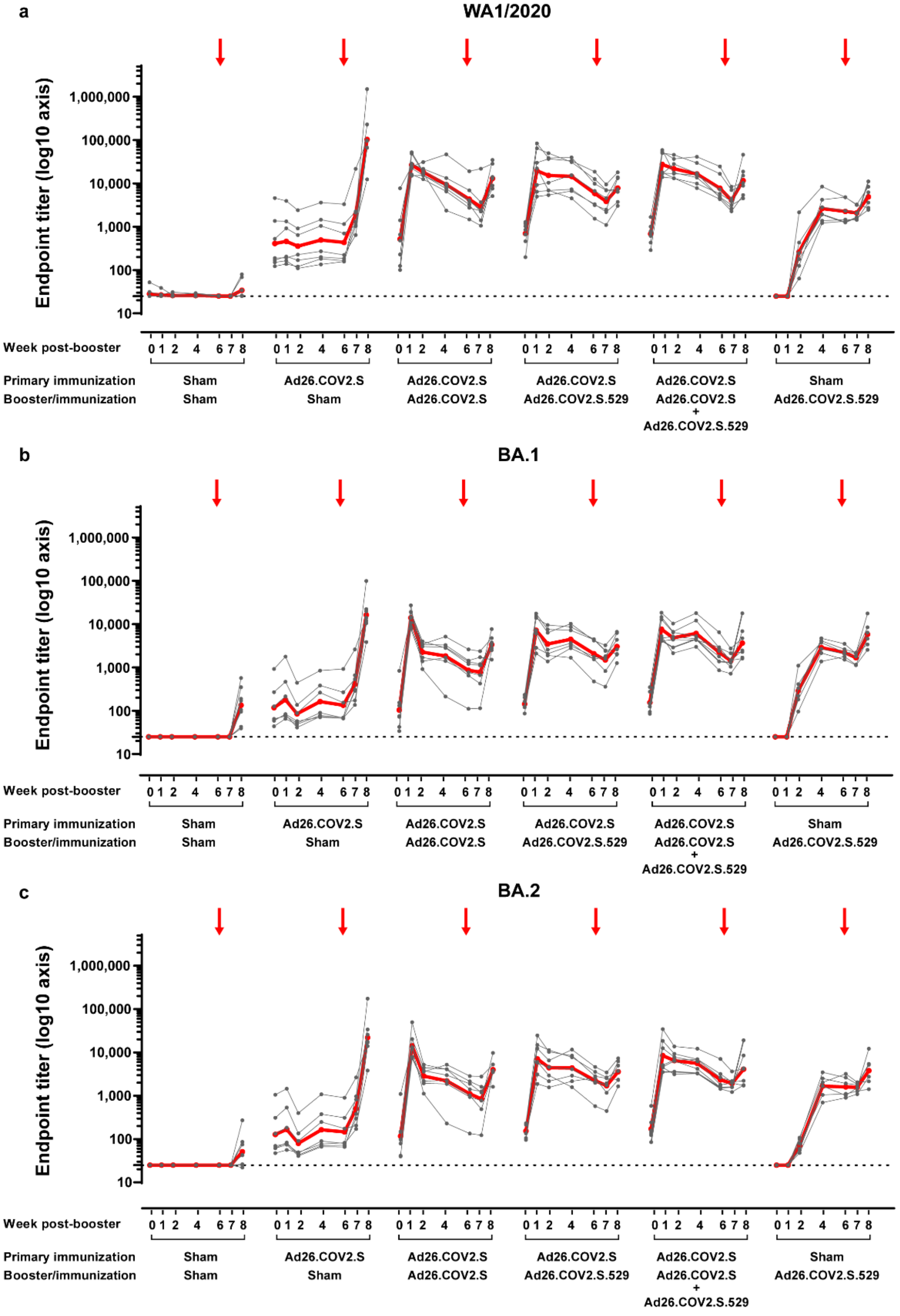

Supplementary Fig. 2. SARS-CoV-2 RBD WA1/2020, BA.1-, and BA.2-specific binding antibody responses to Ad26.COV2.S, Ad26.COV2.S.529, or a combination of Ad26.COV2.S and Ad26.COV2.S.529 late booster vaccination in adult rhesus macaques. SARS-CoV-2 spike RBD

protein–binding antibody concentrations were measured over time in NHP serum samples (42 NHPs and 7 time points; 1 data point not available) with an ELISA using SARS-CoV-2 **a** WA1/2020, **b** BA.1, and **c** BA.2 RBD protein as a coating antigen. Results are expressed as endpoint titers. Antibody levels in the individual animals are depicted with gray points and paired measurements connected with gray lines. The GMT of binding antibody responses per group is indicated with the red line. The horizontal dotted line indicates the LLOD. The red arrows indicate the time of challenge (week 6). For all panels, n=7 animals per group except for the Sham-Ad26.COV2.S.529 group where n=6 animals and for the Sham-Sham group where n=8 animals. Comparisons between specific vaccine groups were made in a Tobit ANOVA with a post hoc z- or t-test. Source data are provided as a Source Data file. ANOVA, analysis of variance; ELISA, enzyme-linked immunosorbent assay; GMT, geometric mean titer; LLOD, lower limit of detection; NHP, nonhuman primate; RBD, receptor-binding domain.

Supplementary Fig. 3

a

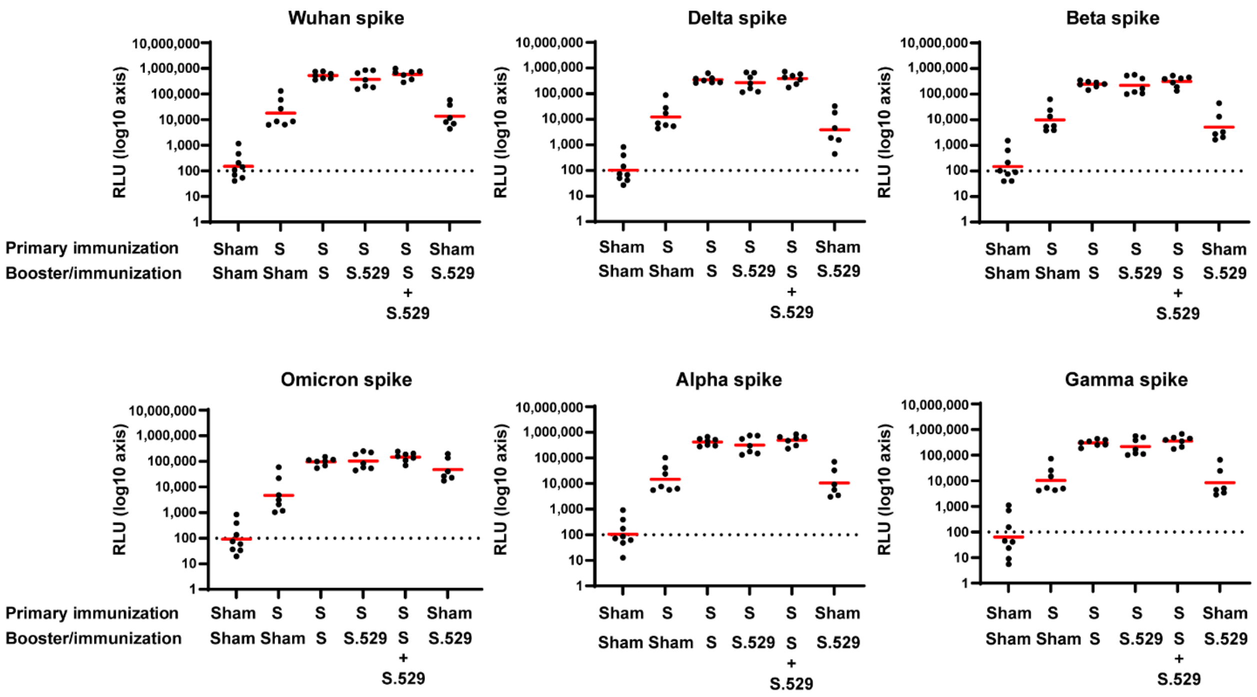

b

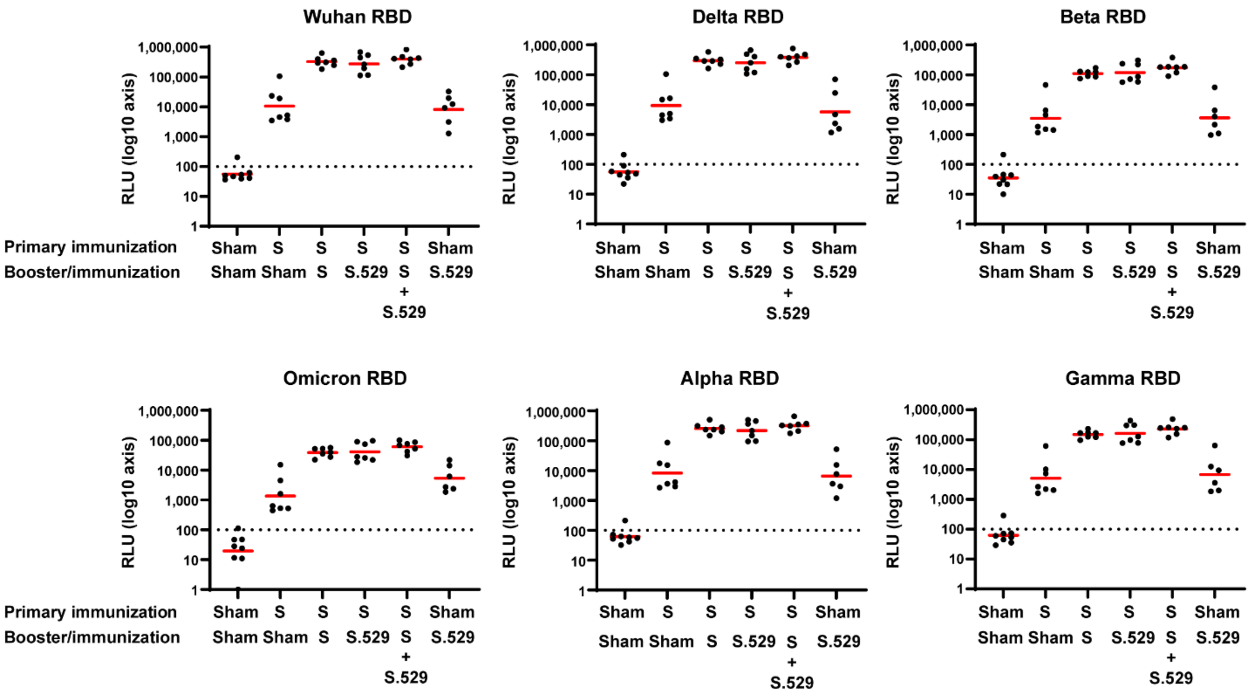

Supplementary Fig. 3. VOC S-specific and RBD-specific binding antibody responses. a S-specific and **b** RBD-specific antibody responses against multiple variants were measured by Meso Scale Discovery

ECLA at week 2 post-vaccination in serum samples from animals boosted with Ad26.COV2.S (indicated as "S" in the figure), Ad26.COV2.S.529 (indicated as "S.529" in the figure), or the mixed vaccine (indicated as "S+S.529" in the figure) and in naïve rhesus macaques immunized with a single dose of Ad26.COV2.S.529. Results are expressed as RLU. The GM RLU is indicated with the horizontal red line. Dotted lines represent the LLOQ. The y-axis was log10 transformed for better visualization. For all panels, n=7 animals per group except for the Sham-S.529 group where n=6 animals and for the Sham-Sham group where n=8 animals. Source data are provided as a Source Data file. ECLA, electrochemiluminescence assay; GM, geometric mean; LLOQ, lower limit of quantification; RBD, receptor-binding domain; RLU, relative light units; S, spike; VOC, variant of concern.

Supplementary Fig. 4

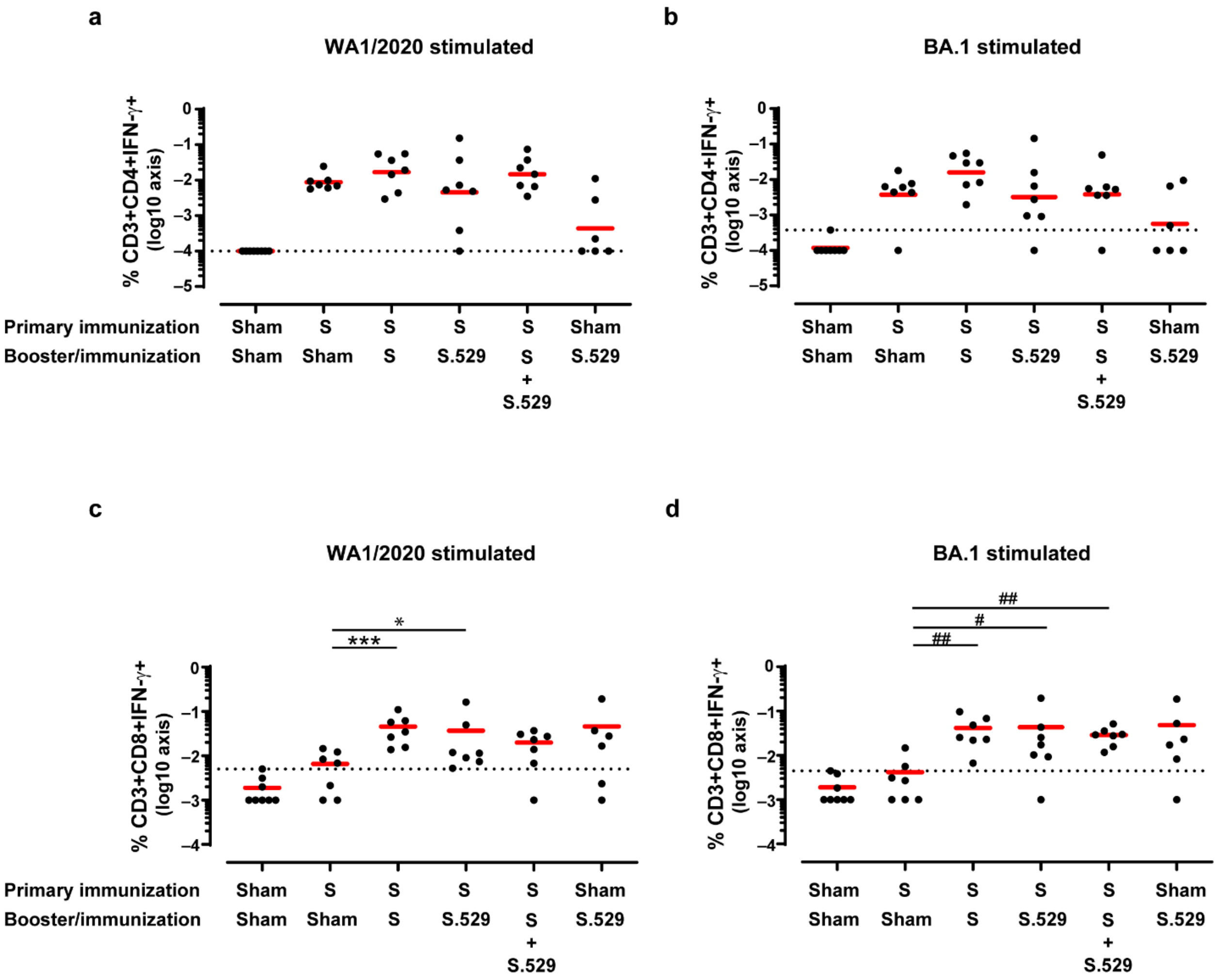

Supplementary Fig. 4. SARS-CoV-2–specific cellular immune responses after Ad26.COV2.S, Ad26.COV2.S.529, or a combination of Ad26.COV2.S and Ad26.CoV2.S.529 late booster in adult rhesus macaques. WA1/2020 (**a** and **c**) and Omicron BA.1 (**b** and **d**) S-specific T-cell responses, as measured in 42 NHP PBMC samples by ICS assays 6 weeks after the booster/immunization. PBMCs were either non-stimulated (i.e., medium stimulated representing background) or stimulated with S WA1/2020 or Omicron BA.1. Background-subtracted frequency of CD8+CD69+ and CD4+CD69+ T cells expressing IFN-γ is depicted. The GM response per group is indicated with the red horizontal line. The dotted line indicates the positivity threshold, calculated as the 95th percentile of sham responses. For panels **a** and **b**, values equal to 0 were imputed to 0.0001 for visualization purposes. For panel **a**, since the 95th percentile of sham responses was equal to 0, the LLOD was also imputed to 0.0001 for visualization purposes. For panels **c**

and **d**, values equal to 0 were imputed to 0.001 for visualization purposes. For all panels, n=7 animals per group except for the Sham-S.529 group where n=6 animals and for the Sham-Sham group where n=8 animals. Comparisons between the sham-immunized group and all other groups with 5-fold Bonferroni adjustment as well as pairwise comparisons between all groups except the control sham-immunized group were made with a 2-sided t-test or exact Mann-Whitney U-test. T-test: *$p<0.05$; ***$p<0.001$. Mann-Whitney U-test: $^{\#}p<0.05$; $^{\#\#}p<0.01$. Non-significant comparisons are not indicated on the graphs. The complete statistical analysis is reported in Supplementary Table 3. Source data are provided as a Source Data file. GM, geometric mean; ICS, intracellular cytokine staining; IFN-γ, interferon gamma; LLOD, lower limit of detection; NHP, nonhuman primate; PBMC, peripheral blood mononuclear cell; S, spike.

Supplementary Fig. 5

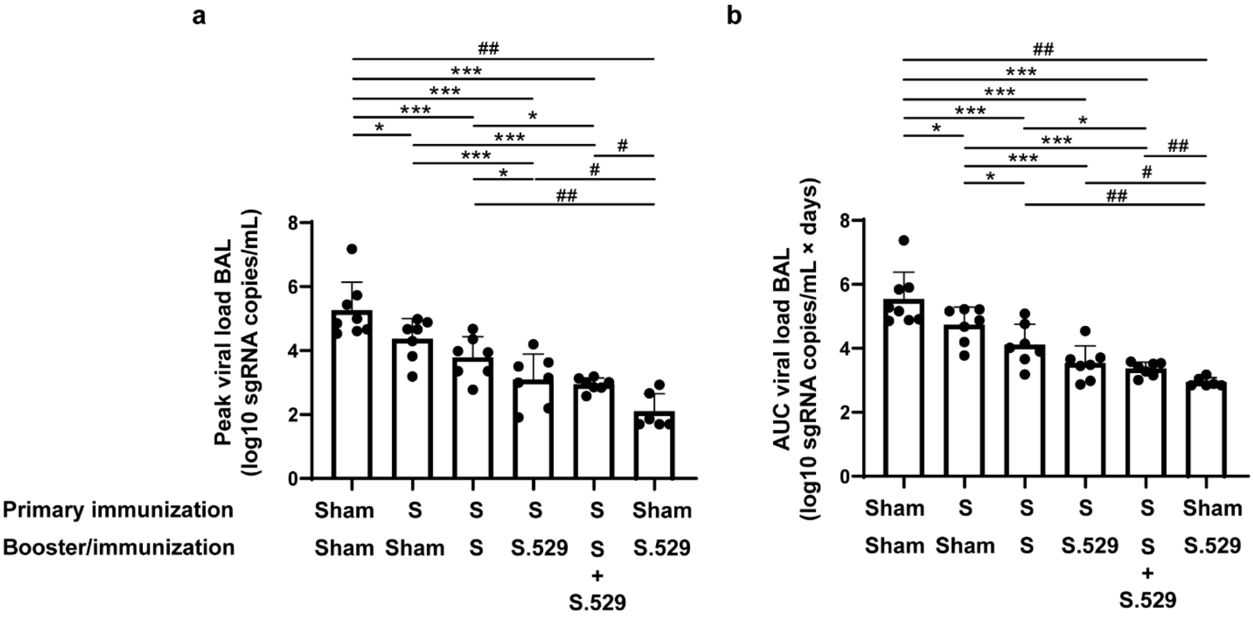

Supplementary Fig. 5. Protective efficacy of the lower respiratory tract against SARS-CoV-2 Omicron BA.1 inoculation after Ad26.COV2.S, Ad26.COV2.S.529, or a combination of Ad26.COV2.S and Ad26.COV2.S.529 late booster in adult rhesus macaques. Animals were challenged with 1×10^6 PFU Omicron BA.1 SARS-CoV-2 administered intranasally and intratracheally 6 weeks after the booster/immunization. **a** Peak viral load in BAL (expressed as sgRNA copies/mL). Peak viral load was defined as the highest viral load measured for each individual NHP over all measurements. **b** Cumulative viral load (sgRNA) in BAL, obtained 7 days prior to challenge, on the day of challenge, 1 day post-challenge-, 2 days post-challenge-, 4 days post-challenge-, 7 days post-challenge, and at sacrifice (day 13 or 14) defined by AUC calculation and expressed as log10 AUC (sgRNA copies/mL × days) from 42 NHPs. Note that for logistical reasons, the sacrifice of the 42 NHPs was split over 2 days, with 3 to 4 NHPs per group sacrificed at day 13 and 3 to 4 NHPs per group sacrificed at day 14. Consequently, for AUC calculation, the day of death of all animals was aligned to day 14 to allow combining data from animals euthanized at day 13 and day 14. Dots represent peak (**a**) and BAL (**b**) viral load for each individual NHP, while bar graphs represent the mean+SD of log-transformed data. For all panels, n=7 animals per group except for the Sham-S.529 group where n=6 animals and for the Sham-Sham group where n=8 animals. Comparisons between the mean BAL AUC values or mean BAL peak viral load values of the sham-immunized group and

all other groups with 5-fold Bonferroni adjustment, as well as pairwise comparisons between all groups except the control sham-immunized group were performed using a Tobit ANOVA with a two-side post hoc z-test or exact Mann-Whitney U-test. Z-test: *p<0.05; ***p<0.001. Mann-Whitney U-test: $^{\#}$p<0.05; $^{\#\#}$p<0.01. Non-significant comparisons are not depicted. Source data are provided as a Source Data file. ANOVA, analysis of variance; AUC, area under the curve; BAL, bronchoalveolar lavage; NHP, nonhuman primate; PFU, plaque-forming units; S, spike; SD, standard deviation; sgRNA, subgenomic RNA.

Supplementary Fig. 6

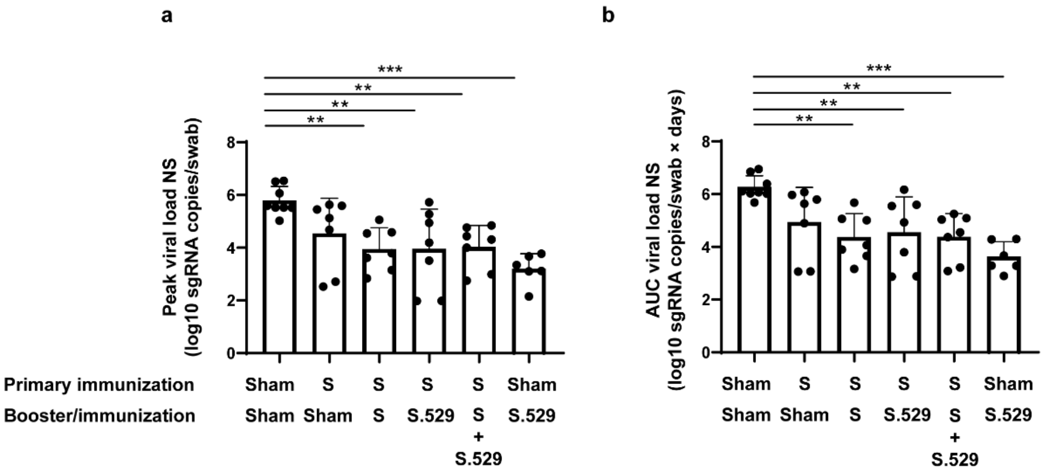

Supplementary Fig. 6. **Protective efficacy of the upper respiratory tract against SARS-CoV-2 Omicron BA.1 inoculation after Ad26.COV2.S, Ad26.COV2.S.529, or a combination of Ad26.COV2.S, Ad26.COV2.S.529 late booster in adult rhesus macaques.** Animals were challenged with 1×10^6 PFU Omicron BA.1 SARS-CoV-2 administered intranasally and intratracheally 6 weeks after the booster/immunization. **a** Peak viral load in NS from 42 NHPs (expressed as sgRNA copies/swab). Peak viral load was defined as the highest viral load measured for each individual NHP over all measurements. **b** Cumulative viral load (sgRNA) in NS, obtained 7 days prior to challenge, on the day of challenge, 1 day, 2 days, 4 days and 7 days post-challenge, and at sacrifice (day 13 or 14) defined by AUC calculation and expressed as log10 AUC (sgRNA copies/swab × days) from 42 NHP. Note that for logistical reasons, the sacrifice of the 42 NHPs was split over 2 days, with 3 to 4 NHPs per group sacrificed at day 13 and 3 to 4 NHPs per group sacrificed at day 14. Consequently, for AUC calculation, the day of death of all animals was aligned to day 14 to allow combining data from animals euthanized at day 13 and day 14. Dots represent peak (**a**) and BAL (**b**) viral load for each individual NHP, while bar graphs represent mean+SD. For all panels, n=7 animals per group except for the Sham-S.529 group where n=6 animals and for the Sham-Sham group where n=8 animals. Comparisons between mean NS AUC values or mean NS peak viral load values of the sham-immunized group and all other groups with 5-fold Bonferroni adjustment, as

well as pairwise comparisons between all groups except the control sham-immunized group were performed using ANOVA with a two-side post hoc t-test. T-test: **$p<0.01$; ***$p<0.001$. Non-significant comparisons are not depicted. Source data are provided as a Source Data file. ANOVA, analysis of variance; AUC, area under the curve; NHP, nonhuman primate; NS, nasal swab; PFU, plaque-forming units; S, spike; SD, standard deviation; sgRNA, subgenomic RNA.

Supplementary Fig. 7

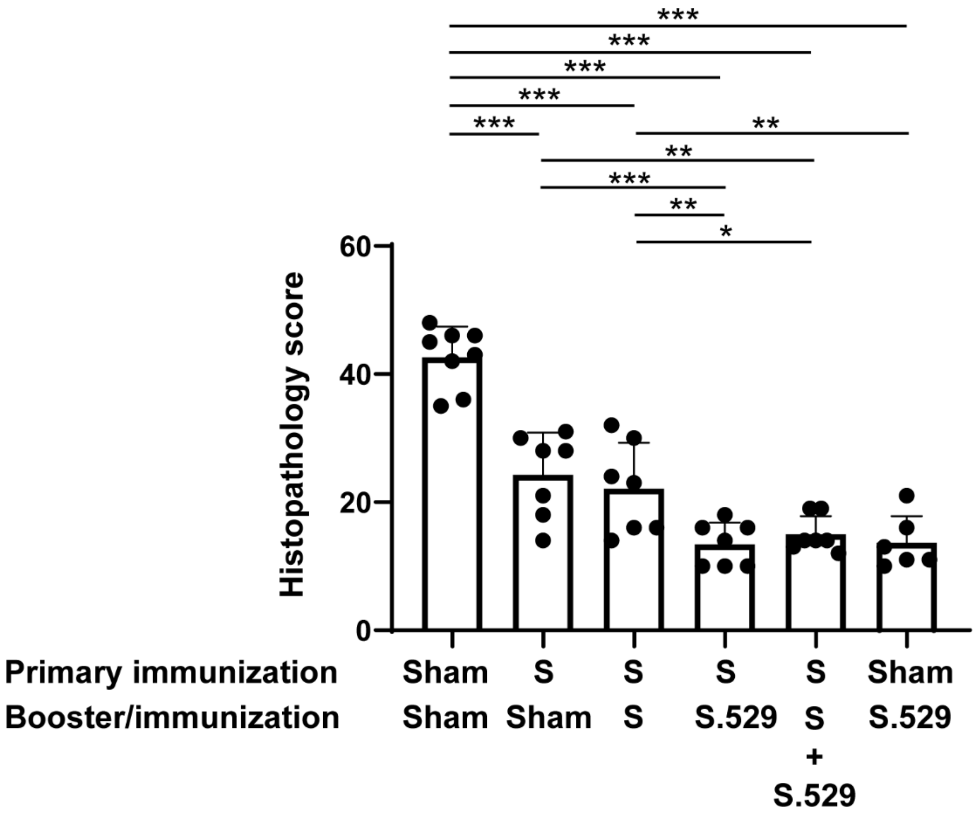

Supplementary Fig. 7. Lung histopathology score after SARS-CoV-2 Omicron BA.1 inoculation after Ad26.COV2.S, Ad26.COV2.S.529, or a combination of Ad26.COV2.S and Ad26.COV2.S.529 late booster in adult rhesus macaques. A detailed microscopic evaluation of lung H&E-stained sections and associated scoring were performed by a pathologist. Seven individual lung lobes were evaluated for each animal in each treatment group (42 NHPs total). Dots represent lung histopathology score for each individual NHP, while bar graphs represent mean+SD. For all panels, n=7 animals per group except for the Sham-S.529 group where n=6 animals and for the Sham-Sham group where n=8 animals. Comparisons between the sham-immunized group and all other groups with 5-fold Bonferroni adjustment as well as pairwise comparisons between all groups except the control sham-immunized group were performed using ANOVA with a post hoc t-test. T-test: *p<0.05; ***p<0.001. Non-significant comparisons are not depicted. Source data are provided as a Source Data file. ANOVA, analysis of variance; H&E, hematoxylin and eosin; NHP, nonhuman primate; S, spike; SD, standard deviation.

Supplementary Fig. 8

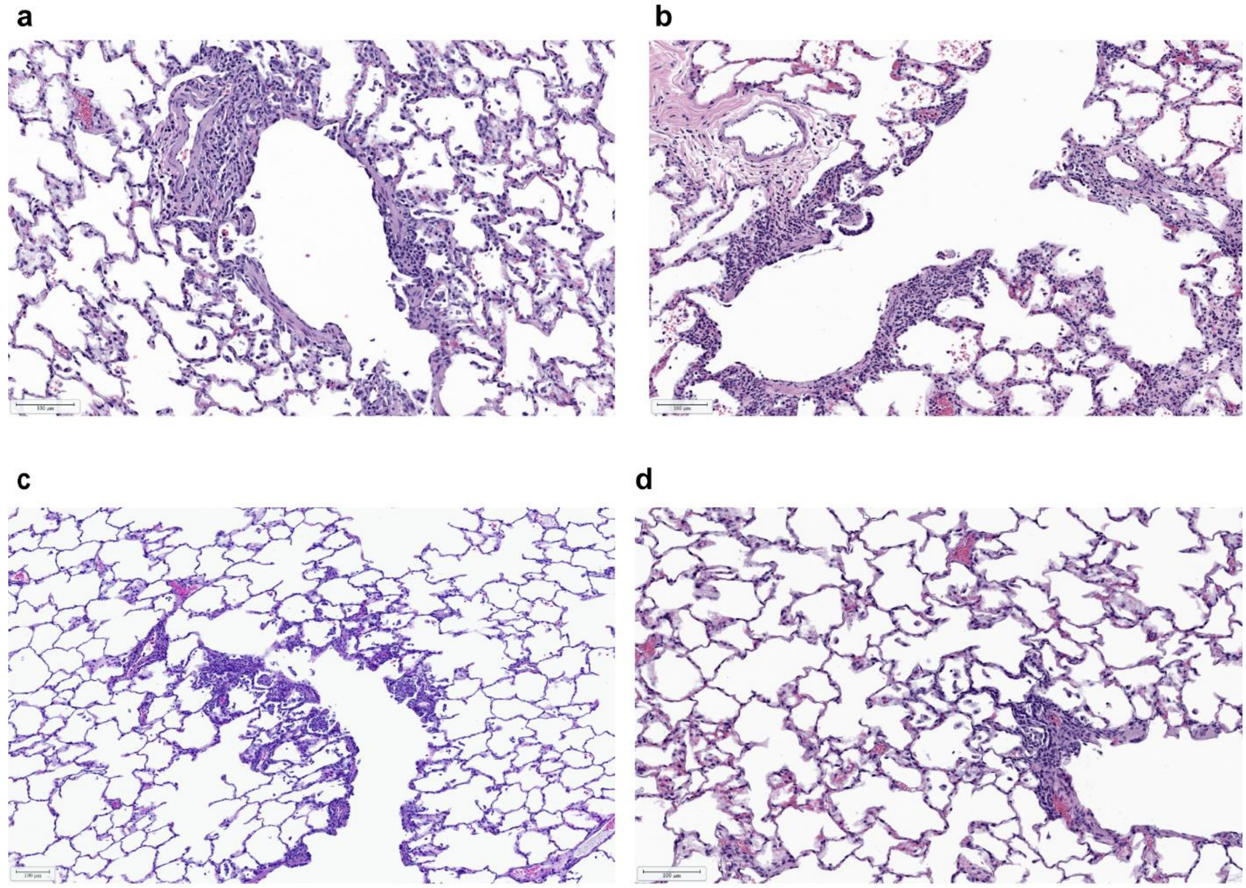

Supplementary Fig. 8. Lung histology after SARS-CoV-2 Omicron BA.1 inoculation after an Ad26.CoV2.S, Ad26.CoV2.S.529, or a combination of Ad26.COV2.S and Ad26.CoV2.S.529 late booster in adult rhesus macaques. Individual lung lobes were evaluated after hematoxylin/eosin staining for each animal in each treatment group (42 NHPs total). 40x magnification overview image. Scale bar is 100 µm or 500 µm. **a** In animals of the sham control group, challenge with Omicron BA.1 was associated with moderate bronchiolar and peribronchiolar inflammation with mild perivascular mononuclear cell infiltrates, and mild alveolar macrophage infiltrates. While all sham control animals had lung lesions consistent with a challenge with the SARS-CoV-2 Omicron variant, only a few **b** Ad26.COV2.S-preimmunized non-boosted animals **c** or that received a booster with Ad26.COV2.S had lesions of comparable severity to those observed in unvaccinated animals, showing **(b)** moderate bronchiolar inflammation characterized by mononuclear and polymorphonuclear cell infiltrates with minimal

peribronchial interstitial inflammation and **(c)** moderate bronchiolar inflammation with minimal alveolar macrophage infiltrates, respectively. **d** The main histological findings in the animals boosted with Ad26.COV2.S.529 (group 4) and the combination of Ad26.COV2.S.529 and Ad26.COV2.S (group 5), and in the naïve animals immunized with 1 dose of Ad26.COV2.S.529 (group 6), were minimal perivascular monocytic infiltrates and peribronchiolar alveolar macrophage infiltrates (representative imagines for groups 4-6).

Supplementary Fig. 9

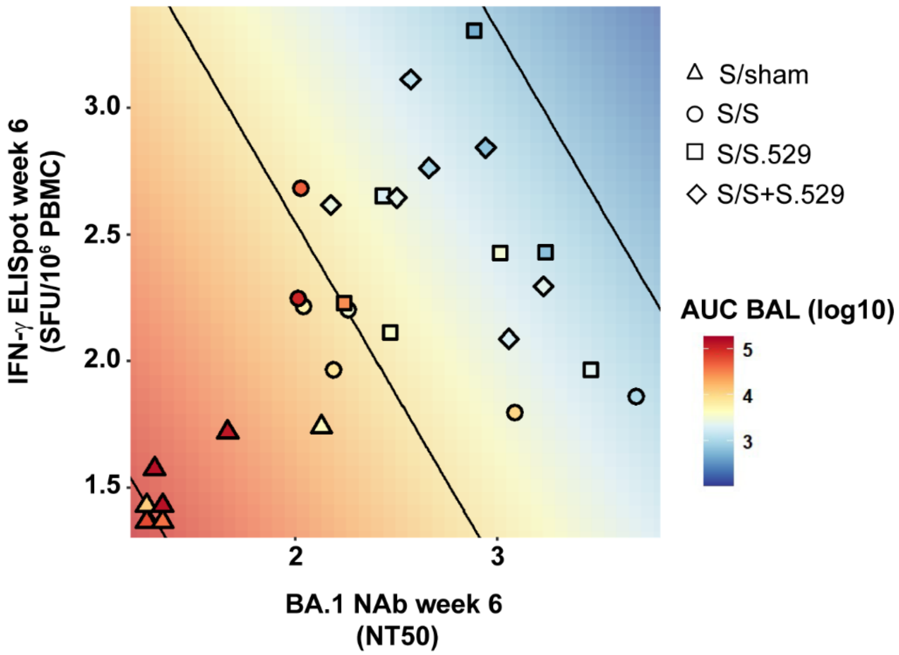

Supplementary Fig. 9. Correlates of protection analysis. Regression of log10 AUC BAL sgRNA on log10 Omicron BA.1 pseudovirus neutralizing antibody titer of all groups having received a primary immunization followed by a late booster. In the figure, the symbols indicate the AUC BAL sgRNA of each NHP as color and their treatment group as shape against a colored background, with contours corresponding to the fitted regression model. n=7 animals per group. Source data are provided as a Source Data file. AUC, area under the curve; BAL, bronchoalveolar lavage; ELISpot, enzyme-linked immunospot assay; IFN-γ, interferon gamma; Nab, neutralizing antibody; NHP, nonhuman primate; NT50, 50% neutralization titer; PBMC, peripheral blood mononuclear cell; S, spike; SFU, spot-forming units; sgRNA, subgenomic RNA.

Supplementary Fig. 10

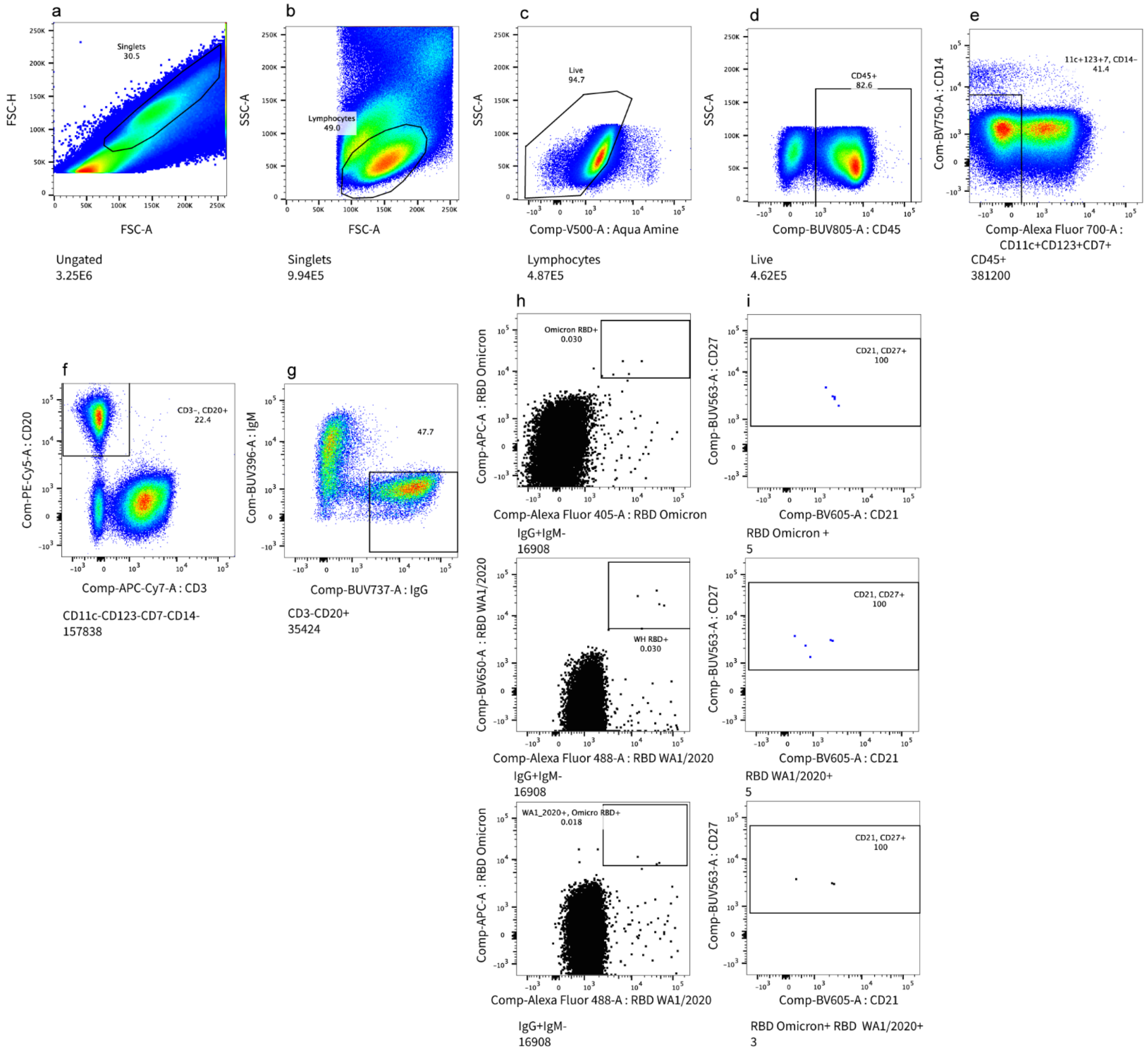

Supplementary Fig. 10. Gating strategy antigen-reactive memory B cells. For analyses, in singlet gate (a), lymphocytes were gated (b) and dead cells were excluded by Aqua dye (c) and CD45 was used as a positive inclusion gate for all leukocytes (d). Within class-switched B cell populations, gated as CD20+IgG+ IgM-CD3-CD14-CD11c-CD123-CD7- (e-g), SARS-CoV-2 WA1/2020 RBD-reactive B cells were identified as

double positive for SARS-CoV-2 (WA1/2020) RBD labeled with different fluorescent probes, and SARS-CoV-2 Omicron BA.1 RBD-reactive B cells were identified as double positive for SARS-CoV-2 (BA.1) RBD proteins labeled with different fluorescent probes (h). Within the antigen-reactive B cells, memory B cells were identified as CD21+ and CD27+ (i).

Supplementary methods

ELISA for Wuhan S–specific binding antibody response after primary vaccination

IgG binding to SARS-CoV-2 spike protein was measured by ELISA using a recombinant spike protein antigen based on the Wuhan Hu-1 SARS-CoV-2 strain (GenBank accession number: MN908947). The SARS-CoV-2 spike protein antigen was adsorbed on 96-well microplates for a minimum of 16 hours at 4°C. Following incubation, plates were washed in phosphate-buffered saline (PBS)/0.05% Tween 20 and blocked with 5% skim milk in PBS/0.05% Tween 20 for 1 hour at room temperature. Serum standards, controls, and NHP serum samples were diluted and incubated on the plates for 1 hour at room temperature. Next, the plates were washed and incubated with peroxidase-conjugated goat antihuman IgG for 1 hour at room temperature, washed, and developed with TMB substrate for 30 minutes at room temperature and protected from light, then stopped with H_2SO_4. The OD was read at 450/620 nm. The antibody concentrations were back calculated on the standard, and the reportable values were generated based on all passing dilutions, expressed in ELISA units [EU]/mL. The LLOD is 3.4 EU/mL, based on the standard lowest interpolation range concentration multiplied per the dilution factor and is used as an informative LLOD. The lower limit of quantification (LLOQ) is based on qualification performed for human samples and has been set at 50.3 EU/mL.

psVNA for Wuhan S–specific neutralizing antibody response after primary vaccination

For assessment of the immunogenicity elicited by Ad26.COV2.S, SARS-CoV-2 spike neutralizing antibody titers were measured by psVNA. Pseudotyped virus particles were made from a modified vesicular stomatitis virus (VSVΔG) backbone and bear the spike glycoprotein of the Wuhan SARS-CoV-2 strain (based on Wuhan Hu-1; GenBank accession number: MN908947). The pseudoparticles contain a luciferase reporter gene used for detection. Serial dilutions of heat-inactivated NHP serum samples were prepared in 96-well transfer plates. The SARS-CoV-2 pseudovirus was added sequentially to the serum dilutions and incubated at 37°C with 5% CO_2 supplementation for 60±5 minutes. Serum-virus complexes were then transferred onto plates previously seeded overnight with Vero E6 cells (ATCC, Cat# CRL-1586) and incubated at 37°C and 5% CO_2 for 20 ± 2 hours. Following this incubation, the luciferase substrate was added to the cells in order to assess the level of luminescence per well. The plates were then read on a luminescence plate reader. The intensity of the luminescence was quantified in relative luminescence units (RLU). The neutralizing titer of a serum sample was calculated as the reciprocal serum dilution corresponding to the 50% neutralization antibody titer (IC50) for that sample. The LLOD is 10, which is the first sample dilution (1:10) used as an informative LLOD. LLOQ is based on qualification performed for human samples and has been set at 33 IC50
