# Supplementary material for: Booster with Ad26.COV2.S or Omicron-adapted vaccine enhanced immunity and efficacy against SARS-CoV-2 Omicron in macaques
Source: Nat Commun. 2023 Apr 7;14:1944. doi: 10.1038/s41467-023-37715-2 (PMC10080532; doi:10.1038/s41467-023-37715-2)
Supplement: Supplementary file 2 — Reporting Summary [file 41467_2023_37715_MOESM2_ESM.pdf]

nature portfolio

Corresponding author(s): Laura Solforosi, Frank Wegmann

Last updated by author(s): Mar 9, 2023

Reporting Summary

Nature Portfolio wishes to improve the reproducibility of the work that we publish. This form provides structure for consistency and transparency in reporting. For further information on Nature Portfolio policies, see our Editorial Policies and the Editorial Policy Checklist.

Statistics

For all statistical analyses, confirm that the following items are present in the figure legend, table legend, main text, or Methods section.

n/a	Confirmed	
☐	☒	The exact sample size (*n*) for each experimental group/condition, given as a discrete number and unit of measurement
☒	☐	A statement on whether measurements were taken from distinct samples or whether the same sample was measured repeatedly
☐	☒	The statistical test(s) used AND whether they are one- or two-sided<br>*Only common tests should be described solely by name; describe more complex techniques in the Methods section.*
☐	☒	A description of all covariates tested
☐	☒	A description of any assumptions or corrections, such as tests of normality and adjustment for multiple comparisons
☐	☒	A full description of the statistical parameters including central tendency (e.g. means) or other basic estimates (e.g. regression coefficient) AND variation (e.g. standard deviation) or associated estimates of uncertainty (e.g. confidence intervals)
☐	☒	For null hypothesis testing, the test statistic (e.g. *F*, *t*, *r*) with confidence intervals, effect sizes, degrees of freedom and *P* value noted<br>*Give P values as exact values whenever suitable.*
☒	☐	For Bayesian analysis, information on the choice of priors and Markov chain Monte Carlo settings
☒	☐	For hierarchical and complex designs, identification of the appropriate level for tests and full reporting of outcomes
☒	☐	Estimates of effect sizes (e.g. Cohen's *d*, Pearson's *r*), indicating how they were calculated

Our web collection on statistics for biologists contains articles on many of the points above.

Software and code

Policy information about availability of computer code

Data collection	No software was used to collect data
Data analysis	Analysis of virologic and immunologic data was performed using GraphPad Prism 9.4.1 (GraphPad Software), FlowJo v10. Statistical analysis was done in SAS 9.4 and R.4.0.2.

For manuscripts utilizing custom algorithms or software that are central to the research but not yet described in published literature, software must be made available to editors and reviewers. We strongly encourage code deposition in a community repository (e.g. GitHub). See the Nature Portfolio guidelines for submitting code & software for further information.

Data

Policy information about availability of data

All manuscripts must include a data availability statement. This statement should provide the following information, where applicable:

- Accession codes, unique identifiers, or web links for publicly available datasets
- A description of any restrictions on data availability
- For clinical datasets or third party data, please ensure that the statement adheres to our policy

All data are available in the manuscript or the supplementary material. Source data are provided with this paper.

Human research participants

Policy information about studies involving human research participants and Sex and Gender in Research.

Reporting on sex and gender	N/A
Population characteristics	N/A
Recruitment	N/A
Ethics oversight	N/A

Note that full information on the approval of the study protocol must also be provided in the manuscript.

Field-specific reporting

Please select the one below that is the best fit for your research. If you are not sure, read the appropriate sections before making your selection.

☒ Life sciences ☐ Behavioural & social sciences ☐ Ecological, evolutionary & environmental sciences

For a reference copy of the document with all sections, see nature.com/documents/nr-reporting-summary-flat.pdf

Life sciences study design

All studies must disclose on these points even when the disclosure is negative.

Sample size	Sample size includes N=34 vaccinated animals (N=6-7 animals for each vaccine group;) and N=8 sham controls. Based on our experience with SARS-CoV-2 in rhesus macaques, this sample size provides power to determine differences in protective efficacy of each vaccinated group compared with the sham controls and between vaccinated groups.
Data exclusions	No data were excluded.
Replication	Virologic and immunologic measures were performed in duplicate and once. Technical replicates were minimally different.
Randomization	Animals were randomized based on original immunization, body weight, age, and SARS-CoV-2 Wuhan spike neutralizing antibody titers and SARS-CoV-2 Wuhan spike binding antibody titers.
Blinding	All immunologic, virologic and histologic assays were performed blinded.

Reporting for specific materials, systems and methods

We require information from authors about some types of materials, experimental systems and methods used in many studies. Here, indicate whether each material, system or method listed is relevant to your study. If you are not sure if a list item applies to your research, read the appropriate section before selecting a response.

Materials & experimental systems

n/a	Involved in the study	
☐	☒	Antibodies
☐	☒	Eukaryotic cell lines
☒	☐	Palaeontology and archaeology
☐	☒	Animals and other organisms
☒	☐	Clinical data
☒	☐	Dual use research of concern

Methods

n/a	Involved in the study	
☒	☐	ChIP-seq
☐	☒	Flow cytometry
☒	☐	MRI-based neuroimaging

Antibodies

Antibodies used

For ELISA and ELISPOT assays anti-macaque IgG HRP (NIH NHP Reagent Resource, Cat# 1b3-HRP; 0320K235 / 070920SC), rabbit polyclonal anti-human IFN-γ (U-Cytech, Cat# CT243).

For ICS assays mAbs against CD279 (clone EH12.1, BB700, BD Biosciences, Cat#566460), CD38 (clone OKT10, PE, NIH NHP Reagent Resource RRID: AB_2819278), CD28 (clone 28.2, PE CY5, BD Biosciences, Cat#555730), CD4 (clone L200,

BV510, BD Biosciences, Cat#563094), CD45 (clone D058-1283, BUV615, BD Biosciences, Cat#751117), CD95 (clone DX2, BUV737, BD Biosciences, Cat#612790), CD8 (clone SK1, BUV805, BD Biosciences, Cat#612889), Ki67 (clone B56, A488, BD Biosciences, Cat#561165), CD69 (clone TP1.55.3, ECD, Beckman Coulter, Cat#6607110), IL10 (clone JES3-9D7, PE CY7, Biolegend, Cat#501420), IL13 (clone JES10-5A2, BV421, BD Biosciences, Cat#563580), TNF-α (clone Mab11, BV650, BD Biosciences, Cat#563418), IL4 (clone MP4-25D2, BV711, BD Biosciences, Cat#564112), IFN-γ (clone B27; BUV395, BD Biosciences, Cat#563563), IL2 (clone MQ1-17H12, APC, BD Biosciences, Cat#554567), CD3 (clone SP34.2, Alexa 700, BD Biosciences, Cat#557917).
For B cell staining mAbs against CD45 (clone D058-1283, BUV805, BD Biosciences, Cat#742055), CD3 (clone SP34.2, APC-Cy7, BD Biosciences, Cat#557757), CD7 (clone M-T701, Alexa700, BD Biosciences, Cat#561603), CD123 (clone 6H6, Alexa700, Biolegend, Cat#306040), CD11c (clone 3.9, Alexa700, Biolegend, Cat#301648), CD20 (clone 2H7, PE-Cy5, BD Biosciences, Cat#555624), IgG (clone G18-145, BUV737, BD Biosciences, Cat#612819), IgM (clone G20-127, BUV395, BD Biosciences, Cat#563903), CD27 (clone M-T271, BUV563, BD Biosciences, Cat#741366), CD21 (clone B-ly4, BV605, BD Biosciences, Cat#740395), CD14 (clone M5E2, Alexa700, Biolegend, Cat#301832), WA1/2020 RBD Biotin (Sino Biological, Cat# 40592-V08B-B) labelled with BV650 streptavidin (BD Bioscience, Cat#563855), WA1/2020 RBD (Sino Biological, Cat # 40592-V08B) labelled with FITC Conjugation Kit (Fast) - Lightning-Link® (Abcam, Cat# ab188285), SARS-CoV-2 B.1.1.529 (Omicron BA.1) RBD (Sino Biological, Cat# 40592-V08H121) labelled with DyLight® 405 Conjugation Kit (Fast) - Lightning-Link® (Abcam, Cat # ab201798) or APC by Alexa Fluor® 647 Conjugation Kit (Fast) - Lightning-Link® (Abcam, Cat # ab269823).

Validation: N/A

Eukaryotic cell lines

Policy information about cell lines and Sex and Gender in Research

Cell line source(s)	HEK293T and Vero E6 commercially purchased (ATCC)
Authentication	All cell lines sourced from ATCC and validated by ATCC
Mycoplasma contamination	Negative for mycoplasma
Commonly misidentified lines (See ICLAC register)	Not used

Animals and other research organisms

Policy information about studies involving animals; ARRIVE guidelines recommended for reporting animal research, and Sex and Gender in Research

Laboratory animals	42 outbred Indian-origin rhesus macaques (Macaca mulatta), 4.8 and 8.7 years old
Wild animals	None
Reporting on sex	36 females and 6 males with 4 males allocated to test group 1, and 2 males allocated to test group 6
Field-collected samples	None
Ethics oversight	Animal experiment approval was provided by the Institutional Animal Care and Use Committee (IACUC) at BIOQUAL, Inc. (Rockville, MD, USA). The test facility is accredited by the American Association for Accreditation of Laboratory Animal Care (AAALAC), and animal experiments were performed in accordance with the standards of the AAALAC International's reference resource: the eighth edition of the Guide for the Care and Use of Laboratory Animals, the Animal Welfare Act as amended, and the 2015 reprint of the Public Health Service Policy on Humane Care and Use of Laboratory Animals.

Note that full information on the approval of the study protocol must also be provided in the manuscript.

Flow Cytometry

Plots

Confirm that:

☒ The axis labels state the marker and fluorochrome used (e.g. CD4-FITC).

☒ The axis scales are clearly visible. Include numbers along axes only for bottom left plot of group (a 'group' is an analysis of identical markers).

☒ All plots are contour plots with outliers or pseudocolor plots.

☒ A numerical value for number of cells or percentage (with statistics) is provided.

Methodology

Sample preparation	10^6 PBMCs/well were re-suspended in 100 µL of R10 media
Instrument	BD FACSymphony

Software	FlowJo v10
Cell population abundance	Cell population abundance shown in each figure
Gating strategy	See Supplementary Fig. 10. Singlet gating of the starting cell population, FSC/SSC Lymphocytes, dead cells were excluded by Aqua dye and CD45 was used as a positive inclusion gate for leukocytes. Within class-switched B-cell population gated as CD20+IgG+IgM-CD3-CD14-CD11c-CD123-CD7-, SARS-CoV-2 WA1/2020 RBD-specific B cells were identified as double positive for SARS-CoV-2 (WA1/2020) RBD labeled with different fluorescent probes, and SARS-CoV-2 (BA.1) RBD-specific B cells are identified as double positive for SARS-CoV-2 (BA.1) RBD proteins labeled with different fluorescent probes.

☒ Tick this box to confirm that a figure exemplifying the gating strategy is provided in the Supplementary Information.
